# Modulating regulated cell death: mechanistic insights into traditional Chinese medicine metabolites for ischemia/reperfusion-induced acute kidney injury

**DOI:** 10.3389/fphar.2026.1779205

**Published:** 2026-07-02

**Authors:** Shaowu Zhang, Jiawen Deng, Tongtong Ma, Jixin Tang, Liping Ou, Peng Wang, Betty Yuen Kwan Law, Huafeng Liu

**Affiliations:** 1 State Key Laboratory of Quality Research in Chinese Medicine, Faculty of Chinese Medicine, Macau University of Science and Technology, Avenida Wai Long, Taipa, Macau, China; 2 Department of Nephrology, National Clinical Key Specialty Construction Program (2023); Institute of Nephrology; Guangdong Provincial Key Laboratory of Autophagy and Major Chronic Non-communicable Diseases; Key Laboratory of Prevention and Management of Chronic Kidney Disease of Zhanjiang City; Affiliated Hospital of Guangdong Medical University, Guangdong Medical University, Zhanjiang, Guangdong, China; 3 Department of Anesthesiology, Affiliated Hospital of Guangdong Medical University, Guangdong Medical University, Zhanjiang, Guangdong, China

**Keywords:** acute kidney injury, ischemia/reperfusion injury, regulated cell death, renoprotection, traditional Chinese medicine metabolites

## Abstract

Acute kidney injury (AKI), characterized by a rapid decline in renal function, represents a significant global health burden due to its high mortality and frequent progression to chronic kidney disease (CKD). Ischemia/reperfusion (I/R) injury is a major cause of AKI, yet clinically effective pharmacotherapies remain elusive. The pathogenesis of I/R-AKI is critically driven by regulated cell death (RCD) pathways—including apoptosis, ferroptosis, pyroptosis, and necroptosis—which mediate tubular epithelial damage and inflammatory responses. Consequently, targeting these RCD pathways emerges as a promising therapeutic strategy. Certain bioactive metabolites found in medicinal plants, including those used in traditional Chinese medicine (TCM), have demonstrated considerable potential in alleviating renal I/R injury. This review provides a detailed analysis of the mechanistic roles of key RCD pathways in I/R-AKI. Furthermore, it synthesizes preclinical evidence over recent decades to illustrate how specific TCM metabolites (e.g., hydroxysafflor yellow A, silibinin, salvianolic acid B, and gypenoside XLIX) confer renoprotection by modulating these RCD processes. To our knowledge, this work is the first to integrate the four major RCD pathways with a spectrum of TCM metabolites that modulate these processes in I/R-AKI. By presenting this integrated perspective, our review highlights TCM as a valuable repository for promising modulators of RCD pathways in I/R-AKI and outlines key priorities for future translational research.

## Graphical Abstract

**Figure:**
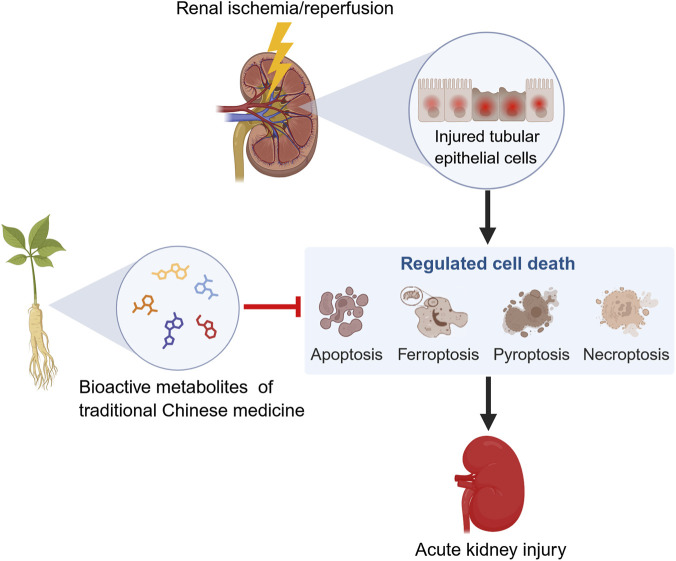

## 1 Introduction

Acute kidney injury (AKI) is a critical clinical syndrome characterized by a rapid decline in renal function, affecting approximately 13 million patients worldwide annually and contributing to roughly 1.7 million deaths each year (Li et al., 2023; Li C. S. Z. et al., 2025). Evidence suggests that patients who survive AKI are at a markedly elevated risk of progressing to chronic kidney disease (CKD), creating further strain on global health systems (Li C. S. Z. et al., 2025). Ischemia/reperfusion (I/R) injury, a major contributor to AKI, occurs in critical conditions (e.g., shock, massive hemorrhage) and surgical procedures (e.g., renal transplantation, cardiovascular operations) (Chen and Yang, 2025). Current AKI treatment strategies mainly involve supportive measures, including hemodynamic optimization and elimination of nephrotoxic exposures. Beyond renal replacement therapy, there are no established medical or surgical treatments for AKI at present (Shi et al., 2023). This significant public health challenge highlights the urgent need to identify new therapeutic targets and develop effective pharmacological treatments for AKI.

The pathological features of I/R-AKI primarily include renal tubular damage, inflammatory responses, and vascular impairment. Notably, the death of tubular epithelial cells (TECs) plays a key role in the development of I/R-AKI (Shi et al., 2023; Li et al., 2024). Although TECs can recover from sublethal damage, cell death leads to irreversible functional impairment. Importantly, dying TECs often release damage-associated molecular patterns (DAMPs), which serve as key initiators and amplifiers of inflammatory responses during tissue injury (Sanz et al., 2023). Cell death can be categorized into accidental cell death (ACD) and regulated cell death (RCD). ACD is an uncontrolled, passive process primarily characterized by unregulated necrosis. In contrast, RCD, which includes apoptosis, ferroptosis, pyroptosis and necroptosis, is governed by specific molecular pathways and may be modulated or inhibited pharmacologically (Noh and Padanilam, 2024). Recent studies have highlighted the growing importance of RCD signaling pathways in I/R-AKI, with accumulating evidence demonstrating their crucial role in renal injury progression (Sanz et al., 2023; Li et al., 2024). Therefore, targeting RCD pathways represents a promising therapeutic strategy for I/R-AKI.

Traditional Chinese medicine (TCM) and its bioactive metabolites have been globally valued for centuries due to their natural origins and therapeutic efficacy. These metabolites have garnered significant attention for their broad therapeutic potential in managing various diseases, including hypertension, diabetes, and liver cancer (Anwanwan et al., 2020; Li X. et al., 2022; Piragine et al., 2022). Importantly, accumulating evidence suggests that specific TCM metabolites may attenuate I/R-AKI progression by targeting RCD pathways in TECs, underscoring their potential as promising therapeutic agents. Therefore, this review examines in detail the mechanisms of key RCD pathways and their contributions to AKI development and progression. Critically, we synthesize the existing evidence demonstrating certain TCM metabolites as effective modulators that alleviate I/R-AKI by inhibiting these RCD processes.

## 2 Literature search and methods

While this manuscript was designed as a narrative rather than a systematic review, a structured literature search was performed to enhance completeness. We searched PubMed from January 2000 to September 2025. The following keyword combinations were employed: (“Acute Kidney Injury” OR “AKI”) AND (“Ischemia-reperfusion” OR “Ischemia/reperfusion” OR “Ischemia reperfusion” OR “I/R” OR “IR” OR “IRI”) AND (“Regulated Cell Death” OR “RCD” OR “Apoptosis” OR “Ferroptosis” OR “Pyroptosis” OR “Necroptosis”). For TCM metabolites, the inclusion criteria focused on studies investigating the modulation of RCD pathways in I/R-AKI by metabolites which TCM contains. We excluded studies on metabolites not associated with TCM; studies that examined crude medicinal species extracts without characterizing the active single metabolite; and conference abstracts, non-research commentaries, and news articles.

All botanical drugs mentioned in this review were taxonomically validated using Plants of the World Online (POWO, https://powo.science.kew.org), with their full names—including authorities and families—provided. The active substances under investigation are single, chemically defined metabolites, not crude extracts or multi-component mixtures. Throughout this review, the term “TCM metabolites” denotes metabolites that are constituents of medicinal species used in TCM; it does not imply that these metabolites are unique to TCM.

## 3 Key RCD pathways in I/R-AKI: mechanisms and detrimental effects

### 3.1 Apoptosis

**FIGURE 1:**
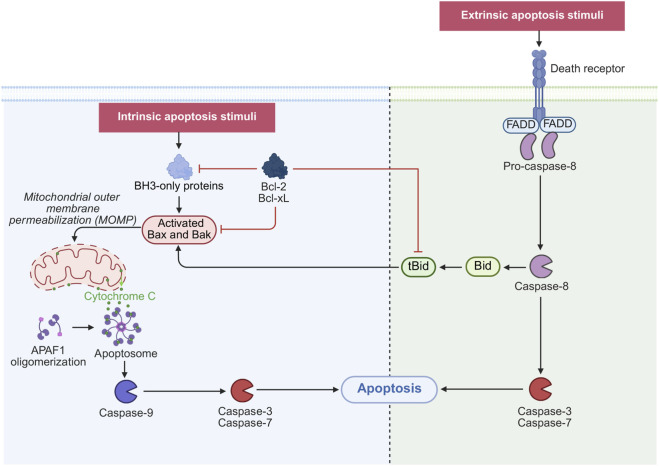
Schematic overview of the intrinsic and extrinsic apoptotic pathways. In the intrinsic (mitochondrial) pathway, cellular stress signals (e.g., DNA damage, oxidative stress) shift the balance of Bcl-2 family proteins, promoting the activation of proapoptotic Bax and Bak. These effector proteins oligomerize to form pores in the mitochondrial outer membrane, a process known as mitochondrial outer membrane permeabilization. This leads to the release of cytochrome C into the cytosol, where it binds to APAF1 and, in the presence of dATP/ATP, triggers the assembly of the apoptosome. This multiprotein complex recruits and activates the initiator caspase-9, which then cleaves and activates the executioner caspases, primarily caspase-3, to dismantle the cell. In the extrinsic (death receptor) pathway, ligation of death receptors (e.g., Fas, TNFR1) by their cognate ligands (e.g., FasL, TNF-α) recruits the adaptor protein FADD and the initiator pro-caspase-8 to form the death-inducing signaling complex (DISC). This leads to caspase-8 activation. Active caspase-8 can directly cleave and activate executioner caspases (e.g., caspase-3). Additionally, it can cleave the BH3-only protein Bid into its active form, tBid, which activates the intrinsic pathway, thereby amplifying the apoptotic signal.

Apoptosis, a well-characterized form of RCD in I/R-AKI, is orchestrated by caspases and defined by hallmark morphological features, including plasma membrane blebbing, cell shrinkage, nuclear changes (pyknosis, karyorrhexis, and fragmentation), and formation of apoptotic bodies that are swiftly phagocytosed by neighboring cells without eliciting inflammation (Havasi and Borkan, 2011; Sanz et al., 2023). In I/R-AKI, both intrinsic and extrinsic pathways converge to execute apoptosis. The intrinsic pathway is triggered by intracellular stressors (particularly mitochondrial oxidative stress and DNA damage), leading to mitochondrial outer membrane permeabilization (MOMP). This process is governed by the Bcl-2 family balance, where proapoptotic proteins (Bax, Bad, Bid) overwhelm antiapoptotic members (Bcl-2, Bcl-xL), ultimately activating caspase-9 and culminating in apoptosis execution primarily through caspase-3 activation. In parallel, the extrinsic pathway is initiated by perturbations of the extracellular microenvironment (e.g., cytokine surges such as Fas ligand and TNF-α) that engage plasma membrane death receptors, transduced through caspase-8 activation, and ultimately converges on the same executioner phase predominantly mediated by caspase-3 (Galluzzi et al., 2018; Sanz et al., 2023). These executioner caspases (primarily caspase-3, with contributions from caspase-7) systematically cleave key cellular substrates, including structural proteins, thereby orchestrating the characteristic apoptotic phenotype marked by cellular dismantling while maintaining membrane integrity (Li et al., 2024). The key mechanisms of apoptosis described above are illustrated in Figure 1.

Critically, TECs are exquisitely vulnerable to these caspase-driven apoptotic cascades, positioning apoptosis as an important driver of I/R-AKI pathophysiology. Apoptotic TEC loss disrupts tubular integrity, causing paracellular “backleak” of solutes and reduced glomerular filtration rate (GFR). Detached apoptotic cells and debris aggregate via β-integrin-mediated interactions, forming obstructive casts that elevate intratubular pressure, further impairing GFR. Additionally, impaired proximal tubular reabsorption enhances solute delivery to distal nephron segments, stimulating tubuloglomerular feedback-mediated vasoconstriction of afferent arterioles and further depressing GFR (Havasi and Borkan, 2011). This creates a vicious cycle where initial apoptotic injury triggers hemodynamic alterations that exacerbate renal dysfunction.

### 3.2 Ferroptosis

While caspase-dependent apoptosis is well-established in I/R-AKI, accumulating evidence highlights ferroptosis as another crucial RCD pathway contributing to tubular injury (Li S. et al., 2022). This iron-dependent cell death is characterized by excessive lipid peroxidation and distinct morphological features including mitochondrial shrinkage, cristae reduction, increased membrane density, outer mitochondrial membrane rupture, and plasma membrane rupture (Pan et al., 2022; Zhu et al., 2024). During I/R-AKI, reperfusion-induced oxidative stress combines with catalytic iron to drive peroxidation of polyunsaturated fatty acid (PUFA)-containing phospholipids through both enzymatic (lipoxygenase-mediated) and non-enzymatic (Fenton reaction) pathways (Braughler et al., 1986; Farmer and Mueller, 2013; Nakamura et al., 2019).

**FIGURE 2:**
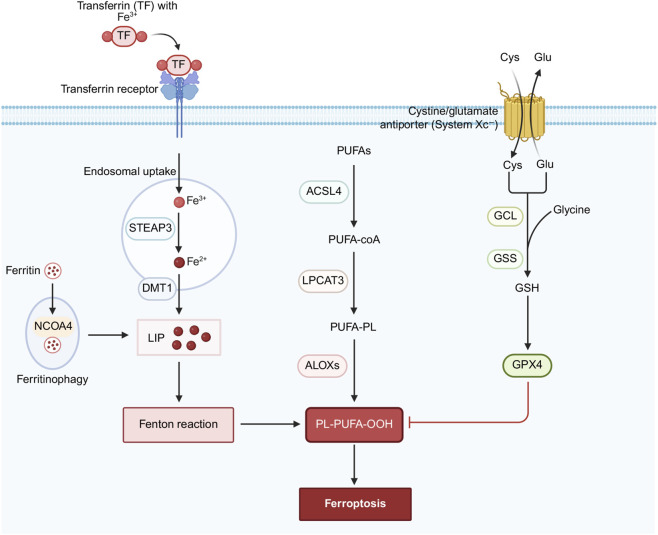
Key molecular mechanisms of ferroptosis. Ferroptosis is executed through three core processes. (1) Iron overload: Transferrin (TF)-bound Fe^3+^ is internalized via transferrin receptor (TFR). Fe^3+^ is reduced to Fe^2+^ by STEAP3 and released into the LIP by DMT1. NCOA4-mediated ferritinophagy further increases LIP. (2) Membrane lipid peroxidation: The LIP drives non-enzymatic lipid peroxidation via the Fenton reaction. In parallel, PUFAs are esterified into membrane phospholipids (PUFA-PLs) by ACSL4 and LPCAT3, which are then oxidized by lipoxygenases (ALOXs) to generate lethal phospholipid hydroperoxides (PL-PUFA-OOH). (3) Antioxidant defense failure: The System Xc^−^ antiporter imports cystine (Cys) for synthesis of GSH by GCL and GSS. GSH is essential for GPX4 to reduce PL-PUFA-OOH into non-toxic alcohols, thereby suppressing ferroptosis. Inhibition of this axis leads to unchecked lipid peroxidation and cell death.

The execution of ferroptosis involves three interconnected core mechanisms: (1) Dysregulation of iron metabolism, primarily through ferritinophagy and subsequent accumulation of the labile iron pool (LIP) (Luo et al., 2025); (2) Amplification of lipid peroxidation, facilitated by acyl-CoA synthetase long-chain family member 4 (ACSL4), which enriches cellular membranes with peroxidation-susceptible PUFAs (Qin et al., 2026); and (3) Inactivation of the key antioxidant enzyme glutathione peroxidase 4 (GPX4), resulting from either TRIM21-mediated ubiquitination or depletion of its essential cofactor glutathione (GSH) (Sun et al., 2023). The underlying molecular mechanisms of ferroptosis are shown in Figure 2. Additionally, cytosolic high-mobility group box 1 (HMGB1) binding to ACSL4 has been shown to promote TEC ferroptosis, as validated in an I/R-AKI model using renal tubule-specific Hmgb1 conditional knockout mice (Zhao et al., 2023). In contrast, global Trim21 deletion has been shown to restore GPX4 protein levels and alleviate ferroptosis in renal tissues of I/R-AKI mice, consistent with the observed TRIM21-dependent ubiquitination of GPX4 in HK-2 cells (Sun et al., 2023). Beyond these pathways, recent studies have identified additional regulatory layers, including the ferroptosis suppressor protein 1 (FSP1)-CoQ10 system, which operates parallel to GPX4 to protect against lipid peroxidation (Bersuker et al., 2019), and dihydroorotate dehydrogenase (DHODH), which mitigates mitochondrial membrane peroxidation (Mao et al., 2021). The anti-ferroptotic function of the FSP1-CoQ10 system in renal tissue has been demonstrated using global Fsp1 knockout mice in I/R-AKI (Tonnus et al., 2021) and renal tubule-specific Fsp1 knockout mice in oxalate-induced AKI (Ye et al., 2025). For DHODH, its protective role against ferroptosis in renal tissue has been supported in cisplatin-induced AKI, where AAV9-mediated DHODH overexpression attenuated ferroptosis (Li B. et al., 2025). However, for both systems, dedicated renal tubule-specific genetic validation in I/R-AKI models is still lacking. A unique and pathologically significant feature of ferroptosis in I/R-AKI is its wave-like propagation, leading to synchronized TEC necrosis (Linkermann et al., 2014; Riegman et al., 2020), a pattern distinct from other forms of regulated necrosis (Sanz et al., 2023).

This synchronized ferroptotic cell death significantly exacerbates renal dysfunction and contributes to AKI pathogenesis through multiple detrimental mechanisms: (1) Plasma membrane rupture directly disrupts tubular integrity, causing backleak of filtrate and promoting cast formation (Linkermann et al., 2014); (2) Electrophiles derived from lipid peroxidation form protein adducts that act as DAMPs, triggering robust pro-inflammatory responses (Bayır et al., 2023; Sanz et al., 2023); and (3) Persistent iron dysregulation sustains lipid peroxidation via the Fenton reaction, exacerbating tubular damage and inflammation, creating a profibrotic microenvironment that may drive AKI progression (Yanatori and Kishi, 2019; Zhao et al., 2023).

Emerging evidence suggests that ferroptosis occurs predominantly during the reperfusion phase (Tang et al., 2021), positioning it as a promising therapeutic target. Pharmacological inhibitors, such as the radical-trapping antioxidants ferrostatin-1 and liproxstatin-1, effectively inhibit lipid peroxidation (Sanz et al., 2023). Furthermore, iron chelators and compounds that bolster cellular antioxidant capacity (e.g., via Nrf2 pathway activation) have demonstrated protective effects in preclinical models (Stockwell et al., 2017). Additionally, inhibiting TRIM21-mediated GPX4 degradation or disrupting the HMGB1–ACSL4 interaction may offer renal protection, given the validated pro-ferroptotic roles of these regulators in I/R-AKI models (Sun et al., 2023; Zhao et al., 2023). Conversely, activating the FSP1–CoQ10 axis or enhancing DHODH activity could also be therapeutically valuable, as these systems have also been implicated in ferroptosis suppression in renal tissue (Tonnus et al., 2021; Li B. et al., 2025; Ye et al., 2025). Collectively, these findings highlight ferroptosis as a critical pathway in I/R-AKI pathophysiology and a viable target for intervention, particularly during the vulnerable reperfusion window.

### 3.3 Pyroptosis

**FIGURE 3:**
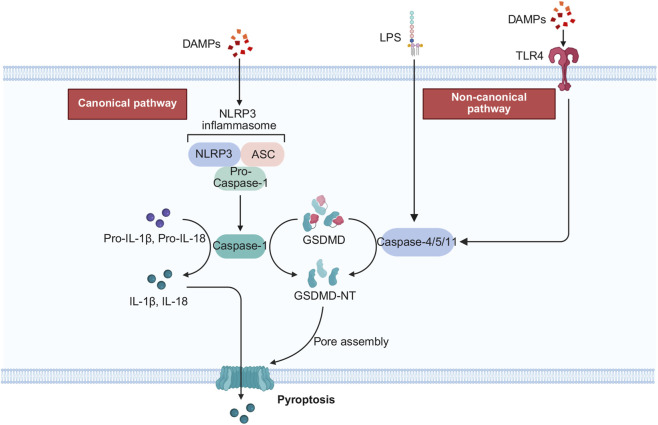
Key molecular mechanisms of pyroptosis. This schematic illustrates the two principal signaling pathways driving pyroptosis. Canonical pathway: DAMPs released during I/R injury activate the NLRP3 inflammasome complex. The assembly of NLRP3, ASC, and pro-caspase-1 leads to caspase-1 activation. Caspase-1 subsequently cleaves both pro-IL-1β/pro-IL-18 into mature cytokines and GSDMD to generate its N-terminal fragments (GSDMD-NT). Non-canonical pathway: DAMPs can also engage this pathway, culminating in the activation of caspase-4/5/11 (caspase-11 in mice), which directly cleaves GSDMD. In both pathways, GSDMD-NT oligomerizes to form pores in the plasma membrane, resulting in the release of inflammatory cytokines (IL-1β, IL-18) and ultimately leading to lytic cell death (pyroptosis).

Pyroptosis is a highly inflammatory form of RCD characterized by gasdermin family-mediated pore formation in the plasma membrane, leading to cellular lysis and release of proinflammatory cytokines (Li T. et al., 2021). This process occurs through two well-defined pathways in renal I/R injury: the canonical pathway (caspase-1-dependent) and non-canonical pathway (caspase-4/5/11-dependent) (Zheng et al., 2022). In the canonical pathway, DAMPs generated during I/R injury (e.g., ROS, mitochondrial DNA) activate pattern recognition receptors such as NLRP3, which then recruits apoptosis-associated speck-like protein containing a CARD (ASC) and pro-caspase-1 to form inflammasomes (Zheng et al., 2022). Activated caspase-1 cleaves both gasdermin D (GSDMD) and pro-IL-1β/pro-IL-18, generating mature cytokines and GSDMD-N-terminal fragments that oligomerize to form membrane pores (Wang Y. et al., 2020). In parallel, the non-canonical pathway in renal I/R injury executes pyroptosis through caspase-4/5/11-mediated cleavage of GSDMD (Li et al., 2024). The canonical and non-canonical signaling pathways of pyroptosis are schematized in Figure 3. Additionally, activated caspase-11 can promote NLRP3 inflammasome activation via the pannexin-1/ATP/P2X7 pathway, thereby connecting the non-canonical and canonical pathways and amplifying the inflammatory response (Zheng et al., 2022).

The role of pyroptosis in I/R-AKI is supported by multiple lines of evidence: (1) significant upregulation of pyroptosis-related proteins (caspase-1, caspase-11, IL-1β) following renal I/R (Yang et al., 2014); (2) endoplasmic reticulum stress (ERS)-mediated activation of the C/EBP homologous protein (CHOP)/caspase-11 pathway in TECs (Yang et al., 2014); and (3) Tisp40-mediated aggravation of GSDMD-dependent pyroptosis through NF-κB pathway activation, as demonstrated by correlated expression patterns and functional validation *in vivo* and *in vitro* (Xiao et al., 2020). Notably, oxidative stress represents a key trigger, with Nrf2/HO-1 signaling pathway serving as an important negative regulator of pyroptosis (Pang et al., 2020). Unexpectedly, recent research has revealed complex context-dependent functions of GSDMD, with one study suggesting a protective role through non-cell autonomous regulation of necroptosis (Tonnus et al., 2022). This complexity may explain contradictory results from different experimental models and highlights the need for further investigation into pyroptosis networks in human AKI.

Therapeutically, targeting pyroptosis has shown promise in preclinical models. Inhibition of NLRP3 with specific inhibitors or siRNA alleviates renal fibrosis (Li H. et al., 2021), while compounds like salvianolic acid B (via Nrf2 activation) (Pang et al., 2020), b-hydroxybutyrate (Tajima et al., 2019), and disulfiram (via caspase-11/GSDMD pathway inhibition) (Cai et al., 2022) have demonstrated protective effects against I/R-AKI through anti-pyroptotic mechanisms. MicroRNAs, particularly miR-92a-3p and miR-302a-3p, also emerge as key regulators of pyroptosis by targeting Nrf1 and FMR1 respectively (Wang R. et al., 2020; Bai et al., 2021).

### 3.4 Necroptosis

**FIGURE 4:**
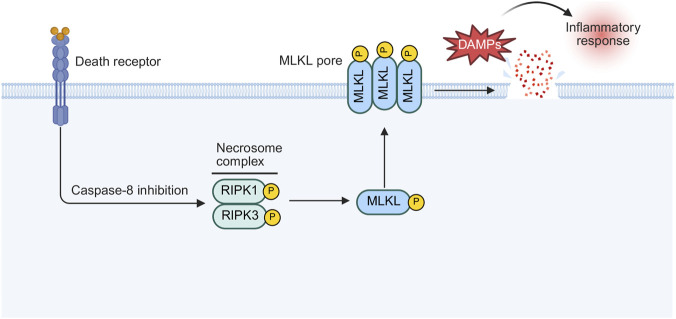
Key molecular mechanisms of necroptosis. This schematic depicts the canonical signaling cascade of necroptosis. The pathway is typically initiated by death receptor (e.g., TNFR1) engagement under conditions where caspase-8 activity is inhibited. This leads to the assembly of the necrosome complex, wherein RIPK1 recruits and interacts with RIPK3 via their reciprocal RHIM domains. RIPK3 then recruits and phosphorylates the terminal effector, MLKL. p-MLKL undergoes oligomerization and translocates to the plasma membrane, where it forms pores that disrupt membrane integrity, culminating in cell lysis and the release of DAMPs.

Necroptosis is a kinase-driven programmed necrotic process characterized by the activation of receptor-interacting serine/threonine-protein kinase one and 3 (RIPK1/RIPK3) and phosphorylation of the executioner protein mixed lineage kinase domain-like pseudokinase (MLKL) (Kolbrink et al., 2023). Morphologically, it resembles unregulated necrosis with rapid plasma membrane disruption, leading to the release of DAMPs and robust inflammation (Li et al., 2024). The canonical signaling cascade of necroptosis is depicted in Figure 4. It is typically initiated by death receptor activation (e.g., TNFR1, Fas). Under conditions of caspase-8 inhibition or deficiency, RIPK1 and RIPK3 oligomerize via RHIM-domain interactions to form the necroptotic signaling complex (necrosome), which recruits and phosphorylates MLKL (Morgan and Kim, 2022). Phosphorylated MLKL oligomerizes and translocates to the plasma membrane, where it mediates necroptotic cell death through multiple mechanisms: triggering calcium influx, externalizing phosphatidylserine, and ultimately compromising plasma membrane integrity (Gong et al., 2017). Furthermore, RIPK3 can translocate to mitochondria and interact with mitofilin, triggering mitochondrial DNA release and activating the cGAS/STING pathway to amplify proinflammatory signaling, thereby exacerbating renal injury (Feng et al., 2022).

Within the context of renal I/R injury, necroptosis significantly contributes to tubular epithelial cell death and inflammation. Substantial evidence indicates activation of this pathway in I/R-AKI, with upregulation of RIPK3/MLKL in experimental models and, importantly, accumulation of p-MLKL observed in necrotic tubules of human AKI patients (Jun et al., 2020; Li et al., 2024). The detrimental impact of necroptosis extends beyond acute tubular injury to drive progression toward chronic kidney disease. Studies utilizing RIPK3- or MLKL-deficient mice demonstrate that inhibiting necroptosis not only mitigates initial kidney damage and inflammation but also confers long-term protection against renal fibrosis following I/R (Chen et al., 2018). This attenuation of fibrotic remodeling is supported by a significant reduction in inflammatory cell infiltration and expression of pro-fibrotic markers, underscoring how necroptosis sustains a persistent inflammatory response that culminates in CKD (Chen et al., 2018). Notably, RIPK3 may also exacerbate injury through necroptosis-independent mechanisms, including direct promotion of inflammatory signaling (Newton et al., 2016; Sanz et al., 2023).

Various therapeutic interventions, including pharmacological inhibitors such as necrostatin-1, as well as TRPC6 activation, extracellular vesicles from stem cells, and recombinant macrophage migration inhibitory factor (MIF), have demonstrated protective effects against necroptosis in experimental AKI (Yuan et al., 2017; Stoppe et al., 2018; Shen et al., 2019). In conclusion, necroptosis represents a significant and multifaceted pathway that critically contributes to the pathogenesis of renal I/R injury and the subsequent progression to fibrosis, highlighting its potential as a therapeutic target for acute and chronic kidney damage.

## 4 Modulation of RCD pathways by TCM metabolites in I/R-AKI

Medicinal species, including those used in TCM, represent a prolific source of structurally diverse bioactive metabolites, driving extensive preclinical investigation into their renoprotective potential against AKI. Previous studies have indicated that certain TCM metabolites exert renoprotective effects by modulating autophagy, suppressing inflammatory responses, and mitigating oxidative stress in the kidneys (Liu Y. et al., 2022; Rui et al., 2022). Recently, there has been growing research interest in the pathways and mechanisms by which TCM metabolites inhibit various forms of RCD, such as apoptosis, ferroptosis, pyroptosis, and necroptosis (Chen et al., 2025). These metabolites show potential as therapeutic agents for I/R-AKI through their ability to modulate RCD pathways.

However, when interpreting preclinical data on TCM metabolites, the potential for pan-assay interference compounds (PAINS) must be considered. Many PAINS can generate false-positive signals in *vitro* assays (Bolz et al., 2021). Among the reviewed TCM metabolites, the following fall into this PAINS category: quercetin, hyperoside, scutellarein, chrysin, diosmetin, tilianin, nicotiflorin, and eriocitrin. To address this issue and facilitate a critical evaluation of the existing evidence, we categorized the strength of support for each metabolite into three tiers: Level A (Robust) includes metabolites whose activity has been validated in at least one mammalian *in vivo* I/R-AKI model and has been supported by rigorous orthogonal mechanistic confirmation (e.g., genetic knockout, specific pathway inhibitors, or target engagement assays), and which are not classified as known PAINS; Level B (Moderate) comprises metabolites whose activity has been confirmed in at least one mammalian *in vivo* I/R-AKI model, but without rigorous orthogonal mechanistic validation, or metabolites that are recognized PAINS with *in vivo* support; Level C (Limited/High risk) applies to metabolites for which evidence is derived exclusively from *in vitro* cell line experiments or which are recognized PAINS lacking *in vivo* validation. Conclusions from Level C studies should be interpreted with caution. This tiered classification underscores that stand-alone *in vitro* findings have limited translational relevance to the complex *in vivo* pathophysiology. An overview of the research findings on TCM metabolites that modulate the RCD pathways in I/R-AKI is summarized in Table 1. Detailed experimental parameters, including *in vivo* and *in vitro* models, dosing regimens, controls, treatment durations, and the lowest protective doses or concentrations tested, are provided in Supplementary Table S1.

**TABLE 1:** Overview of TCM metabolites modulating RCD pathways in I/R-AKI.

Metabolite	Structure	Representative source	Experiment objects	RCD	Functional targets	References	Evidence level
Loganin	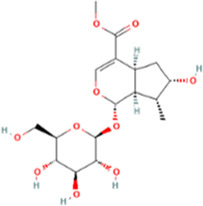	*Cornus officinalis* Siebold and Zucc. (Cornaceae)	Mice; NRK-52E cells	Apoptosis	Inhibited Bax/cleaved caspase-3; Upregulated Bcl-2; Suppressed JAK2/STAT3	Huang et al. (2022)	B
Astragaloside IV	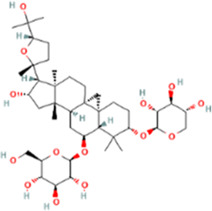	*Astragalus mongholicus* Bunge (Fabaceae)	Rats	Apoptosis	Inhibited Bax/cleaved caspase-3; Upregulated Bcl-2; Inhibited p38 MAPK	Gui et al. (2013), Su et al. (2022)	B
Emodin	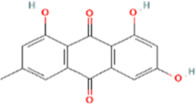	*Rheum palmatum* L. (Polygonaceae)	Mice; HK-2 cells	Apoptosis	Inhibited p53 pathway; Downregulated cleaved caspase-3; Upregulated Bcl-2	Wang et al. (2022b), Lu et al. (2023)	A
Cordycepin	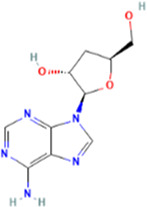	*Ophiocordyceps sinensis* (Berk.) G.H. Sung et al. (Ophiocordycipitaceae)	Rats	Apoptosis	Inhibited mitochondrial apoptosis; Downregulated Cleaved caspase-3 and -9	Han et al. (2020)	B
Ligustrazine	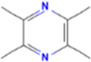	*Oreocome striata* (DC.) Pimenov and Kljuykov (Apiaceae)	Rats; Mice	Apoptosis	Inhibited caspase-3/cleaved caspase-3; Upregulated Bcl-2	Feng et al. (2004), Feng et al. (2011), Jiang et al. (2020)	B
Berberine	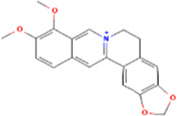	*Coptis chinensis* Franch. (Ranunculaceae)	Rats; TECs	Apoptosis	Inhibited mitochondrial apoptosis; Activated Sirt1 to inhibit p53	Yu et al. (2013), Xie et al. (2017), Lin et al. (2018)	A
Cryptotanshinone	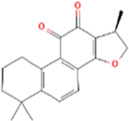	*Salvia miltiorrhiza* Bunge (Lamiaceae)	Mice; HK-2 cells	Apoptosis	Inhibited p38 MAPK; Modulated Bax/Bcl-2 and caspase-3	Bai et al. (2019), Zhu et al. (2019)	A
Hyperoside	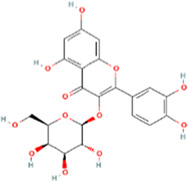	*Abelmoschus manihot* (L.) Medik. (Malvaceae)	Mice; HK-2 cells	Apoptosis	Inhibited mitochondrial fission and caspase-3 cleavage	Wu et al. (2019)	B
Hydroxysafflor yellow A	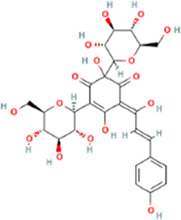	*Carthamus tinctorius* L. (Asteraceae)	Mice; HK-2 cells	Apoptosis	Inhibited CCR4/Ca^2+^/MAPK pathways; Modulated Bcl-2/Caspase-3	Wang et al. (2022a), Wang et al. (2025)	A
Notoginsenoside R1	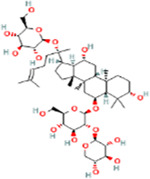	*Panax notoginseng* (Burkill) F.H.Chen (Araliaceae)	Rats	Apoptosis	Upregulated Bcl-2; Inhibited p38/NF-κB axis	Liu et al. (2010)	B
Polydatin	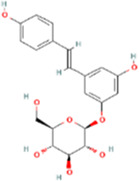	*Reynoutria japonica* Houtt. (Polygonaceae)	Mice; TECs	Apoptosis	Activated Shh/Gli1 pathway; Upregulated Bcl-2; Inhibited caspase-3	Meng et al. (2016)	A
Alisol B 23-acetate	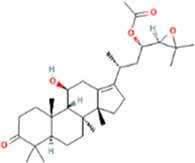	*Alisma plantago-aquatica* subsp. *Orientale* (Sam.) Sam. (Alismataceae)	Mice; mTECs	Apoptosis	Activated FXR; Downregulated Bax/Cleaved caspase-3; Upregulated Bcl-2	Luan et al. (2021)	A
Tetramethylpyrazine	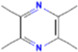	*Oreocome striata* (DC.) Pimenov and Kljuykov (Apiaceae)	Rats; NRK cells	Apoptosis	Inhibited tubular apoptosis and cleaved caspase-3	Sun et al. (2020)	B
β-elemene	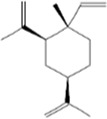	*Curcuma aromatica* Salisb. (Zingiberaceae)	Mice; NRK-52E cells	Apoptosis	Inhibited TLR4/MyD88 signal; Modulated Bax/Bcl-2 and cleaved caspase-3	Gong et al. (2025)	A
Madecassoside	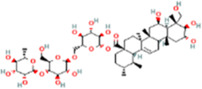	*Centella asiatica* (L.) Urb. (Apiaceae)	Mice; TECs	Apoptosis	Inhibited JNK/c-JUN pathway; Modulated Bax/Bcl-2 and caspase-3	Shan et al. (2024)	A
Tilianin	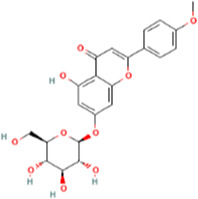	*Agastache rugosa* (Fisch. and C.A.Mey.) Kuntze (Lamiaceae)	Mice	Apoptosis	Inhibited ERK/EGR1 axis; Upregulated Bcl-2/Bcl-xL; Downregulated Bax/Bad/caspase-3	Liu Z. et al. (2022)	B
Scutellarein	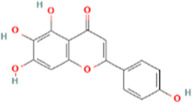	*Scutellaria barbata* D.Don (Lamiaceae)	Rats; HK-2 cells	Apoptosis	Promoted COX-2 degradation; Inhibited caspase-3/PARP cleavage	Liu et al. (2021)	B
Nicotiflorin	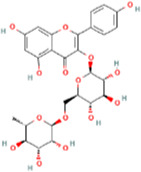	*Nymphaea candida* C.Presl (Nymphaeaceae)	Mice; HK-2 cells	Apoptosis	Activated ATF3 pathway; Upregulated Bcl-2; Downregulated caspase-3	Wang L. et al. (2021)	B
Eriocitrin	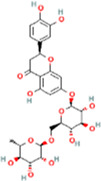	*Citrus limon* (L.) Osbeck (Rutaceae)	Rats; HK-2 cells	Apoptosis	Modulated Bax/Bcl-2 and caspase-3	Xu et al. (2021)	B
Ellagic acid	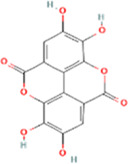	*Punica granatum* L. (Lythraceae)	Rats; NRK-52E cells	Apoptosis	Modulated Bax/Bcl-2 and caspase-3	Liu et al. (2020)	A
Sesamin	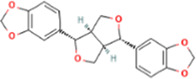	*Sesamum indicum* L. (Pedaliaceae)	Mice	Apoptosis	Promoting CD39-adenosine-A2AR pathway; Inhibited caspase-3	Li et al. (2016)	A
Aloperine	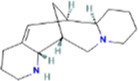	*Sophora alopecuroides* L. (Fabaceae)	Mice; HK-2 cells	Apoptosis	Enhanced AP-1 activity; Upregulated Bcl-2; Downregulated caspase-3	Hu et al. (2016)	A
Paeoniflorin	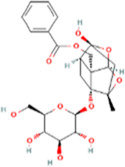	*Paeonia lactiflora* Pall. (Paeoniaceae)	HK-2 cells	Apoptosis	Modulated Bax/Bcl-2	Xing et al. (2023)	C
Neferine	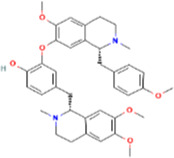	*Nelumbo nucifera* Gaertn. (Nelumbonaceae)	Mice; NRK-52E cells	Apoptosis	Upregulated Klotho	Li et al. (2019)	B
Schisandrin B	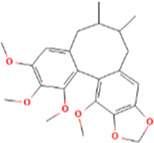	*Schisandra chinensis* (Turcz.) Baill. (Schisandraceae)	Mice; HK-2 cells	Apoptosis	Regulated mitochondrial dynamics; Activated AKT1	Xu et al. (2025)	A
Resveratrol	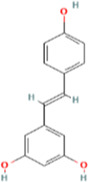	*Reynoutria japonica* Houtt. (Polygonaceae)	Rats	Apoptosis	Inhibited caspase-3; Upregulated Bcl-2 and antioxidants	Alaasam et al. (2024)	B
Costunolide	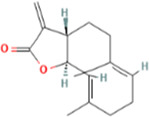	*Dolomiaea costus* (Falc.) Kasana and A.K.Pandey (Asteraceae)	Rats	Apoptosis	Inhibited caspase-3	Güler et al. (2023)	B
Ganoderic Acids	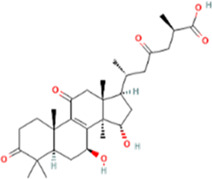	*Ganoderma sichuanense* J.D.Zhao and X.Q.Zhang (Polyporaceae)	Mice; NRK-52E cells	Apoptosis	Decreased the ratios of cleaved caspase-8 and cleaved caspase-3.	Shao et al. (2021)	B
Taraxasterol	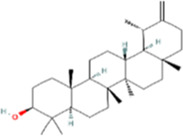	*Taraxacum* F.H.Wigg. (Asteraceae)	Mice; HK-2 cells	Apoptosis	Modulated Bax/Bcl-2	Li et al. (2020)	B
Chrysin	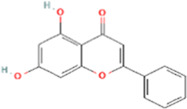	*Oroxylum indicum* (L.) Kurz (Bignoniaceae)	Mice	Apoptosis	Modulated Bax/Bcl-2 and caspase-3	Xu et al. (2019)	B
Quercetin	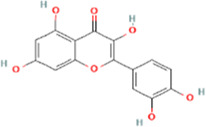	*Styphnolobium japonicum* (L.) Schott (Fabaceae)	Rats	Apoptosis	Inhibited apoptosis-associated factors	Bagheri et al. (2023)	B
Diosmetin	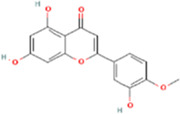	*Galium verum* L. (Rubiaceae)	Mice	Apoptosis	Inhibited mitochondrial apoptosis pathways	Yang et al. (2017)	B
Oleanolic acid	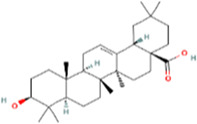	*Ligustrum lucidum* W.T.Aiton (Oleaceae)	Rats	Apoptosis	Inhibited caspase-3	Long et al. (2016)	A
Mangiferin	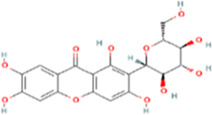	*Anemarrhena asphodeloides* Bunge (Asparagaceae)	Mice	Apoptosis	Inhibited active caspase-3 expression	Wang et al. (2015)	B
Quercetin	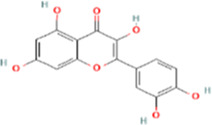	*Styphnolobium japonicum* (L.) Schott (Fabaceae)	Mice; NRK-52E/HK-2 cells	Ferroptosis	Inhibited ATF3; Upregulated SLC7A11 and GPX4	Wang Y. et al. (2021)	B
Paeoniflorin	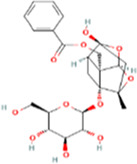	*Paeonia lactiflora* Pall. (Paeoniaceae)	Mice; HK-2 cells	Ferroptosis	Upregulated SLC7A11, enhancing GSH/GPX4 axis	Ma et al. (2023)	A
Pachymic acid	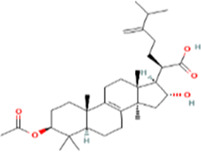	*Wolfiporia hoelen* (Fr.) Y.C.Dai and V.Papp (Fomitopsidaceae)	Mice	Ferroptosis	Upregulated GPX4, SLC7A11, HO-1	Jiang et al. (2021)	B
Cyanidin-3-glucoside	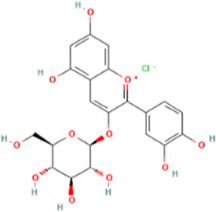	*Morus alba* L. (Moraceae)	Mice; HK-2 cells	Ferroptosis	Activated AMPK pathway; Upregulated GPX4; Downregulated ACSL4	Du et al. (2023)	A
Salidroside	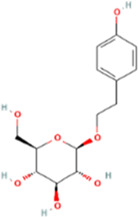	*Rhodiola rosea* L. (Crassulaceae)	Rats; NRK-52E cells	Ferroptosis	Activated PI3K/AKT pathway; Upregulated GPX4; Downregulated ACSL4	Tang et al. (2023b)	A
Vaccarin	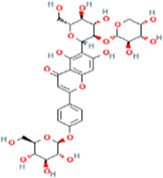	*Gypsophila vaccaria* (L.) Sm. (Caryophyllaceae)	Mice; mTECs	Ferroptosis	Upregulated GPX4 and SLC7A11; Reduced lipid peroxidation	Fan et al. (2025)	A
Echinocystic acid	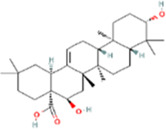	*Gleditsia sinensis* Lam. (Fabaceae)	Neonatal rats	Ferroptosis	Activated Nrf2/GPX4 pathway; Reduced iron overload and lipid ROS	Dang et al. (2025)	A
Loureirin C	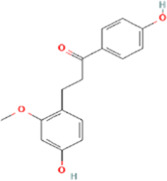	*Dracaena angustifolia* (Medik.) Roxb. (Asparagaceae)	Mice; HK-2 cells	Ferroptosis	Activated Nrf2/HO-1 axis; Upregulated GPX4 and SLC7A11	Qi et al. (2024)	A
Xanthohumol	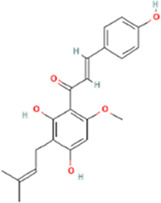	*Humulus lupulus* L. (Cannabaceae)	Rats; HK-2 cells	Ferroptosis	Activated Nrf2/HO-1 pathway; Upregulated GPX4; Downregulated ACSL4	Tang et al. (2023a)	A
Silibinin	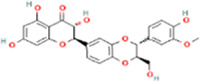	*Silybum marianum* (L.) Gaertn (Asteraceae)	Mice; NRK-52E/HK-2 cell	Ferroptosis	Bound to FTH1 and inhibited ferritinophagy	Deng et al. (2024)	A
Gypenoside XVII	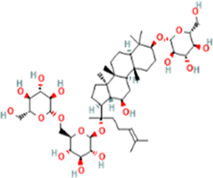	*Gynostemma pentaphyllum* (Thunb.) Makino (Cucurbitaceae)	Mice	Pyroptosis	Inhibited ERS (CHOP) and NLRP3/caspase-1/GSDMD pathway	Wang et al. (2024)	B
Salvianolic acid B	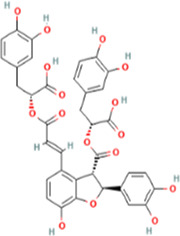	*Salvia miltiorrhiza* Bunge (Lamiaceae)	Mice; HK-2 cells	Pyroptosis	Activated Nrf2 pathway; Inhibited NLRP3 inflammasome and GSDMD cleavage	Pang et al. (2020)	A
Berberine	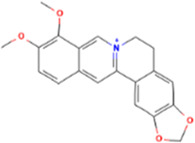	*Coptis chinensis* Franch. (Ranunculaceae)	HK-2 cells	Pyroptosis	Activated FOXO3a/ARC pathway; Downregulated NLRP3, caspase-1, GSDMD-N	Wang and Huang (2025)	C
Naringenin	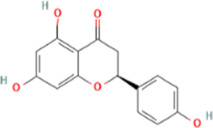	*Citrus maxima* (Burm.) Merr. (Rutaceae)	Mice; HK-2 cells	Pyroptosis	Activated Nrf2/HO-1; Inhibited ERS and NLRP3/caspase-1/GSDMD pathway	Zhang et al. (2022)	A
Parthenolide	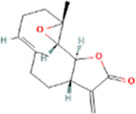	*Magnolia biondii* Pamp. (Magnoliaceae)	TCMK-1 cells	Pyroptosis	Inhibited NF-κB pathway and GSDMD-mediated pyroptosis	Xiao et al. (2020)	C
Aurantiamide	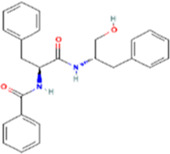	*Portulaca oleracea* L. (Portulacaceae)	Mice; HK-2 cells	Necroptosis	Antagonized GRPR; Inhibited RIPK1/RIPK3/MLKL phosphorylation	He et al. (2024)	A
Gypenoside XLIX	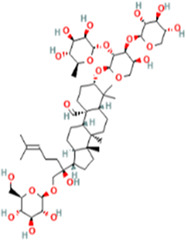	*Gynostemma pentaphyllum* (Thunb.) Makino (Cucurbitaceae)	Mice	Necroptosis	Inhibited IGFBP7/IGF1R axis and RIPK1/RIPK3/MLKL pathway	Yang et al. (2021)	A

All chemical structure images shown in this table were sourced from the PubChem database (https://pubchem.ncbi.nlm.nih.gov).

### 4.1 Modulation of apoptotic pathway

#### 4.1.1 Loganin

Loganin, an iridoid glycoside found in medicinal plants including *Cornus officinalis* Siebold and Zucc. (Cornaceae), has demonstrated significant anti-apoptotic effects in I/R-AKI. In a study by Huang et al., loganin treatment effectively attenuated hypoxia/reoxygenation (H/R)-induced apoptosis in renal tubular epithelial cells (NRK-52E), as evidenced by enhanced cell viability and reduced apoptotic rates in a dose-dependent manner. Mechanistically, loganin downregulated pro-apoptotic proteins (Bax and Cleaved Caspase-3) and upregulated the anti-apoptotic protein Bcl-2. Furthermore, loganin suppressed the activation of the JAK2/STAT3 signaling pathway, which is known to promote inflammation and apoptosis, while simultaneously activating the Nrf2/HO-1 antioxidant pathway. These effects were corroborated in an *in vivo* I/R-AKI mouse model, where loganin administration ameliorated renal tubular damage and improved renal function, highlighting its potential as a therapeutic agent for mitigating apoptosis in I/R-AKI (Huang et al., 2022).

#### 4.1.2 Astragaloside IV

Astragaloside IV (AS-IV), a bioactive saponin isolated from medicinal plants like *Astragalus mongholicus* Bunge (Fabaceae), exerts significant anti-apoptotic effects in I/R-AKI models. Studies indicated that AS-IV pretreatment markedly attenuated renal dysfunction and tubular damage by suppressing oxidative stress and apoptosis. Specifically, AS-IV reduced the expression of pro-apoptotic proteins such as Bax and cleaved caspase-3, while upregulating the anti-apoptotic protein Bcl-2, thereby restoring the mitochondrial apoptotic balance (Gui et al., 2013; Su et al., 2022). Furthermore, AS-IV activated the Keap1-Nrf2/HO-1 signaling pathway, enhancing antioxidant defenses by increasing superoxide dismutase (SOD) activity and reducing malondialdehyde (MDA) levels, which collectively mitigated oxidative stress-induced apoptosis (Su et al., 2022). Additionally, AS-IV inhibited the phosphorylation of p38 MAPK, a key stress kinase involved in apoptosis signaling, further contributing to its renoprotective effects (Gui et al., 2013). These mechanisms were consistent across both ischemia-reperfusion and contrast-induced AKI models, highlighting AS-IV’s potential as a multi-target therapeutic agent for preventing apoptosis in I/R-AKI.

#### 4.1.3 Emodin

Emodin, a bioactive anthraquinone found in medicinal plants like *Rheum palmatum* L. (Polygonaceae), has been shown to mitigate renal apoptosis following I/R-AKI via diverse molecular pathways. According to Wang et al., emodin pretreatment markedly reduced mitochondrial fission and apoptosis in both murine I/R models and human renal tubular epithelial cells (HK-2) under H/R by inhibiting the CAMKII/DRP1 pathway. Specifically, emodin suppressed CAMKII activation and subsequent phosphorylation of DRP1 at Ser616, thereby preserving mitochondrial dynamics, reducing ROS production, and decreasing cytochrome C release and caspase-3 activation ⁠(Wang et al., 2022b). Complementarily, Lu et al. employed network pharmacology and experimental validation to show that emodin exerts its anti-apoptotic effect primarily via the p53 pathway. Their results indicated that emodin downregulated p53 expression, reduced cleaved-caspase-3 and pro-caspase-9 levels, and upregulated Bcl-2, thereby inhibiting tubular epithelial cell apoptosis in both I/R and vancomycin-induced AKI models⁠ (Lu et al., 2023). Together, these studies highlight emodin’s dual mechanisms targeting both mitochondrial fission regulation and p53-mediated apoptotic signaling, underscoring its potential as a multi-target agent against I/R-AKI.

#### 4.1.4 Cordycepin

Cordycepin, a natural adenosine analogue which can be isolated from *Ophiocordyceps sinensis* (Berk.) G.H. Sung et al. (Ophiocordycipitaceae), exhibits notable renoprotection in I/R injury models by inhibiting apoptotic pathways. According to Han et al., pretreatment with cordycepin markedly attenuated renal tubular apoptosis in a rat I/R model, as evidenced by decreased TUNEL-positive staining in renal TECs. Mechanistically, cordycepin administration significantly reduced the activation of key apoptotic executers, cleaved caspase-3 and cleaved caspase-9, indicating suppression of the mitochondrial-mediated intrinsic apoptosis pathway⁠ (Han et al., 2020). These findings underscore the inhibition of apoptosis as a key mechanism through which cordycepin mitigates renal damage, positioning it as a promising therapeutic candidate for I/R-AKI.

#### 4.1.5 Ligustrazine

Ligustrazine, a bioactive alkaloid found in medicinal plants such as *Oreocome striata* (DC.) Pimenov and Kljuykov (Apiaceae), has been shown to significantly reduce apoptosis in experimental models of renal I/R injury. In a rat model of I/R-AKI, ligustrazine treatment markedly reduced renal cell apoptosis, as evidenced by decreased TUNEL-positive cells and suppression of caspase-3/cleaved caspase-3 expression (Jiang et al., 2020). Similarly, Feng et al. reported that ligustrazine pretreatment in mice subjected to renal warm I/R injury significantly attenuated tubular apoptosis, accompanied by reduced oxidative stress (decreased MDA levels and increased SOD activity) and inhibition of neutrophil infiltration (Feng et al., 2011). Furthermore, ligustrazine was shown to upregulate the anti-apoptotic protein Bcl-2 in renal tissues, suggesting a mechanism involving enhanced cellular resistance to apoptotic stimuli (Feng et al., 2004). These findings collectively indicate that ligustrazine protects against I/R-induced renal apoptosis through multiple pathways, including oxidative stress reduction, inflammation suppression, and modulation of apoptosis-related proteins.

#### 4.1.6 Berberine

Berberine (BBR), a bioactive isoquinoline alkaloid abundant in medicinal plants such as *Coptis chinensis* Franch. (Ranunculaceae), exerts potent anti-apoptotic effects in renal I/R injury by targeting multiple molecular pathways. *In vitro* studies using HK-2 cells revealed that BBR pretreatment attenuated H/R-induced apoptosis by suppressing both mitochondrial and ERS pathways, as evidenced by a reduced Bax/Bcl-2 ratio, decreased cytochrome C release, and inhibited caspase-3 activation, along with downregulated expression of ER stress markers GRP78 and CHOP (Yu et al., 2013). Similarly, *in vivo* studies in rat models of renal ischemia-reperfusion (RIR) confirmed that BBR treatment ameliorated renal dysfunction and apoptosis by downregulating Bax, caspase-3, and TNF-α mRNA expression, while upregulating Bcl-2, which in turn helped to preserve mitochondrial integrity and reduce oxidative stress (Visnagri et al., 2015). To enhance its bioavailability, BBR nanoparticles were developed and shown to exert superior protective effects compared to free BBR, further reducing oxidative stress (MDA, ROS), mitochondrial cytochrome C release, and caspase-3 activation in RIR models (Xie et al., 2017). Moreover, BBR was found to activate Sirt1, leading to deacetylation and inhibition of p53, thereby suppressing its nuclear translocation and the subsequent PUMA-mediated mitochondrial apoptosis pathway in rat renal NRK-52E cells under H/R conditions (Lin et al., 2018). Collectively, these findings position BBR as a promising therapeutic agent against I/R-AKI, functioning through multi-target inhibition of key cell death pathways.

#### 4.1.7 Cryptotanshinone

Cryptotanshinone (CTS), a natural metabolite found in several medicinal plants, notably *Salvia miltiorrhiza* Bunge (Lamiaceae), has been demonstrated to significantly mitigate renal apoptosis in renal I/R injury models. In mice with bilateral renal I/R, CTS pretreatment (10 mg/kg/d, 7 days) improved renal function and reduced tubular damage, accompanied by decreased TUNEL-positive cells and cleaved caspase-3 expression. It also suppressed the mitochondrial apoptotic pathway via downregulation of Bax and upregulation of Bcl-2, while inhibiting p38 MAPK phosphorylation (Bai et al., 2019). In H/R-stimulated HK-2 cells, CTS enhanced cell viability, reduced ROS, and increased SOD and CAT activities. Apoptosis was inhibited through reduced Bax and caspase-3 activity and increased Bcl-2. CTS also activated the PI3K/Akt pathway, and its protective effects were reversed by PI3K inhibitor LY294002, indicating PI3K/Akt involvement (Zhu et al., 2019). These findings underscore the role of CTS in attenuating oxidative stress and apoptosis in I/R-AKI.

#### 4.1.8 Hyperoside

Hyperoside, a flavonoid isolated from medicinal plants including *Abelmoschus manihot* (L.) Medik. (Malvaceae), protects against renal I/R injury by attenuating apoptosis and modulating mitochondrial dynamics. According to a study by Wu et al., pretreatment with hyperoside in mice reduced tubular apoptosis, as shown by decreased TUNEL-positive cells and caspase-3 cleavage. It inhibited mitochondrial fission by suppressing OMA1-mediated OPA1 proteolysis, preserving mitochondrial integrity and reducing oxidative stress. Consistent *in vitro*, hyperoside suppressed CoCl_2_-induced apoptosis, fission, and ROS in HK-2 cells via the OMA1-OPA1 axis (Wu et al., 2019). Thus, hyperoside exerts renoprotection by targeting mitochondrial fission-mediated apoptosis and oxidative stress, highlighting its therapeutic potential for I/R-AKI.

#### 4.1.9 Hydroxysafflor yellow A

Hydroxysafflor yellow A (HSYA), a major bioactive metabolite isolated from *Carthamus tinctorius* L. (Asteraceae), has demonstrated significant anti-apoptotic effects in renal I/R injury through multiple molecular pathways. Bai et al. revealed that in rats, HSYA pretreatment significantly improved renal function, reduced tubular damage, and decreased TUNEL-positive cells. Furthermore, HSYA downregulated the expression of caspase-3 and pro-inflammatory cytokines such as TNF-α and IL-1β, while upregulating the anti-inflammatory cytokine IL-10. These effects were associated with inhibition of the TLR4/NF-κB signaling pathway, as evidenced by reduced phosphorylation of IKKβ and IκBα, and decreased nuclear translocation of NF-κB p65 (Bai et al., 2018). Wang et al. further demonstrated that HSYA alleviated renal I/R injury by activating the Akt/GSK-3β/Fyn-Nrf2 axis, which enhanced antioxidant capacity, reduced oxidative stress, and inhibited apoptosis via modulation of Bcl-2 and cleaved caspase-3 expression (Wang et al., 2022a). More recently, Wang et al. identified CCR4 as a novel target of HSYA, showing that it directly bound to CCR4 and inhibited the downstream PLCβ/IP3R/Ca^2+^/MAPK pathway, thereby maintaining calcium homeostasis and reducing p38/JNK-mediated apoptosis in renal TECs (Wang et al., 2025). Together, these studies highlight HSYA as a multi-target agent against apoptosis in I/R-AKI, acting through anti-inflammatory, antioxidant, and calcium-stabilizing mechanisms.

#### 4.1.10 Notoginsenoside R1

Notoginsenoside R1 (NR1), a bioactive saponin found in medicinal plants, notably *Panax notoginseng* (Burkill) F.H.Chen (Araliaceae), has been shown to attenuate renal apoptosis in a rat I/R-AKI model. Pretreatment with NR1 significantly reduced serum creatinine (SCr) levels and improved renal histology, indicating functional recovery. Its antiapoptotic effect was evidenced by decreased TUNEL-positive cells and upregulated Bcl-2 expression, without altering Bax levels. NR1 also suppressed phosphorylation of p38 MAPK and activation of NF-κB—key regulators of inflammatory and apoptotic pathways. Thus, NR1 protects against renal I/R injury by modulating the Bcl-2/Bax balance and inhibiting proapoptotic signaling through the p38/NF-κB axis (Liu et al., 2010).

#### 4.1.11 Polydatin

Polydatin, a stilbenoid glucoside found in medicinal plants including *Reynoutria japonica* Houtt. (Polygonaceae), demonstrates significant anti-apoptotic effects in renal I/R injury by activating the Sonic hedgehog (Shh) signaling pathway (Meng et al., 2016). In that study, polydatin treatment in a murine model of I/R-AKI significantly reduced tubular cell apoptosis, as evidenced by decreased TUNEL-positive cells and lower caspase-3 activity. Furthermore, it upregulated the expression of the anti-apoptotic protein Bcl-2. Mechanistically, polydatin was shown to enhance the secretion of Shh ligand and promote the nuclear translocation of the transcription factor Gli1, a key effector of the Shh pathway. Critically, the administration of cyclopamine (a Smoothened inhibitor) or 5E1 (an anti-Shh antibody) abolished these anti-apoptotic effects, confirming that the nephroprotection conferred by polydatin is dependent on Shh pathway activation.

#### 4.1.12 Alisol B 23-acetate

Alisol B 23-acetate (ABA), a bioactive triterpenoid isolated from *Alisma plantago-aquatica* subsp. *orientale* (Sam.) Sam. (Alismataceae), protects against renal I/R injury by activating Farnesoid X receptor (FXR) and inhibiting tubular cell apoptosis (Luan et al., 2021). In that study, ABA treatment significantly reduced TUNEL-positive cells and caspase-3 activity in mouse kidneys after I/R. As an FXR agonist, ABA promotes FXR nuclear translocation and transcriptional activity. This activation led to the downregulation of Bax and cleaved caspase-3, and the upregulation of Bcl-2. The renoprotective effects were abolished in FXR knockout mice, confirming FXR dependence. Thus, ABA ameliorates I/R-AKI through FXR-dependent mechanisms, with apoptosis modulation being a key component.

#### 4.1.13 Tetramethylpyrazine

Tetramethylpyrazine (TMP), a bioactive metabolite found in medicinal plants, notably *Oreocome striata* (DC.) Pimenov and Kljuykov (Apiaceae), inhibits apoptosis in renal I/R injury. Sun et al. demonstrated that TMP treatment significantly reduced renal tubular apoptosis, suppressed cleaved caspase-3 expression, and improved renal function by lowering SCr and blood urea nitrogen (BUN) levels (Sun et al., 2020). These findings highlight TMP’s role as an anti-apoptotic agent in I/R-AKI.

#### 4.1.14 β-elemene

β-Elemene (ELE), a natural sesquiterpene found in medicinal plants such as *Curcuma aromatica* Salisb. (Zingiberaceae), exhibits potent anti-apoptotic properties against I/R-AKI. Gong et al. reported that ELE pretreatment markedly reduced renal tubular apoptosis, as indicated by decreased TUNEL-positive cells and downregulation of cleaved caspase-3 expression in both a mouse I/R model and H_2_O_2_-stimulated NRK52E cells. Additionally, ELE treatment suppressed the Bax/Bcl-2 ratio, a key indicator of mitochondrial apoptotic pathway activation. Mechanistically, ELE inhibited the TLR4/MyD88/NF-κB/MAPK signaling axis, thereby attenuating inflammation and apoptosis associated with renal I/R injury (Gong et al., 2025). These results underscore the potential of ELE as a therapeutic agent targeting apoptosis in I/R-AKI.

#### 4.1.15 Madecassoside

In the context of I/R-AKI, Madecassoside (MA), a triterpenoid saponin isolated from *Centella asiatica* (L.) Urb. (Apiaceae), has been demonstrated to attenuate renal apoptosis through inhibition of the JNK/c-JUN signaling pathway (Shan et al., 2024). Their study showed that, using both *in vitro* H/R models and *in vivo* renal I/R injury models, MA pretreatment significantly reduced tubular cell apoptosis, as evidenced by decreased expression of Bax and cleaved caspase-3, and increased expression of Bcl-2. Mechanistically, RNA-sequencing and molecular docking analyses revealed that MA directly binds to JNK kinase, inhibiting its phosphorylation and reducing the activation and expression of the downstream transcription factor c-JUN, ultimately leading to suppression of apoptosis-related signaling. Furthermore, MA demonstrated superior renoprotective efficacy compared to the JNK inhibitor SP600125 in experimental I/R-AKI models.

#### 4.1.16 Tilianin

Tilianin, a flavonoid glycoside isolated from medicinal plants including *Agastache rugosa* (Fisch. and C.A.Mey.) Kuntze (Lamiaceae), protects against renal I/R-AKI by modulating the mitochondrial apoptotic pathway (Liu Z. et al., 2022). Their results showed that in a mouse model of I/R-AKI, tilianin administration significantly improved renal function, as evidenced by reduced SCr and BUN levels. Its treatment also markedly reduced tubular injury and apoptosis, indicated by lower TUNEL-positive cell counts and suppressed release of cytochrome C. Mechanistically, tilianin inhibited the phosphorylation of ERK1/2, which led to downregulation of the transcription factor EGR1 and subsequently altered the balance of Bcl-2 family proteins by enhancing the expression of Bcl-2 and Bcl-xL, while reducing Bax, Bad, and caspase-3. Bioinformatic analysis and molecular docking further supported that tilianin potentially targets the ERK/EGR1/Bcl-xL axis, highlighting its role as a natural metabolite capable of attenuating apoptosis in I/R-AKI.

#### 4.1.17 Scutellarein

Scutellarein, a bioactive flavonoid isolated from medicinal plants such as *Scutellaria barbata* D. Don (Lamiaceae), has demonstrated protective effects against apoptosis in I/R-AKI models. In a rat model of I/R-AKI, pretreatment with scutellarein significantly reduced serum levels of BUN and SCr, attenuated tubular injury, and decreased apoptosis as evidenced by TUNEL staining and reduced cleavage of caspase-3 and PARP. In H/R-treated HK-2 cells, scutellarein dose-dependently attenuated the viability loss, LDH release, ROS generation, and apoptosis. Mechanistically, scutellarein targeted COX-2 protein, without affecting its mRNA expression, and promoted its degradation via enhanced autophagy, thereby mitigating inflammation and apoptosis. Overexpression of COX-2 partially reversed the protective effects of scutellarein, confirming COX-2 as a key mediator in its mechanism of action (Liu et al., 2021). These findings establish scutellarein as a promising TCM metabolite for attenuating apoptotic pathways in I/R-AKI.

#### 4.1.18 Nicotiflorin

Nicotiflorin, a flavonol glycoside found in medicinal plants, notably *Nymphaea candida* C. Presl (Nymphaeaceae), protects against renal I/R injury by attenuating apoptosis via the activating transcription factor 3 (ATF3) pathway (Wang L. et al., 2021). The study demonstrated that nicotiflorin pretreatment in mice significantly lowered SCr, BUN, and KIM-1 levels and improved renal histology. It downregulated caspase-3 and Bad while upregulating Bcl-2 and Cyr61. In H/R-treated HK-2 cells, nicotiflorin reduced TUNEL-positive cells and caspase-3 activity. Molecular docking indicated strong binding between nicotiflorin and ATF3, and ATF3 knockdown diminished its anti-apoptotic effects, confirming ATF3-dependent action. Thus, nicotiflorin represents a promising anti-apoptotic agent for I/R-AKI.

#### 4.1.19 Eriocitrin

Eriocitrin, a flavonoid found in various plants, notably *Citrus limon* (L.) Osbeck (Rutaceae), shows anti-apoptotic efficacy in I/R-AKI (Xu et al., 2021). The study revealed that eriocitrin reduced apoptosis in both H/R-induced HK-2 cells and a rat I/R-AKI model. The anti-apoptotic effect was evidenced by decreased expression of Bax and cleaved caspase-3, increased anti-apoptotic protein Bcl-2, and reduced apoptotic rate via flow cytometry. Mechanistically, eriocitrin upregulated dual-specificity phosphatase 14 (DUSP14), which enhanced Nrf2 expression and suppressed NF-κB activation, thereby attenuating oxidative stress and inflammation—key drivers of apoptosis in I/R injury. Inhibition of DUSP14 reversed these protective effects, confirming its critical role in eriocitrin-mediated apoptosis modulation.

#### 4.1.20 Ellagic acid

Ellagic acid, a natural polyphenol found in various medicinal plants, notably *Punica granatum* L. (Lythraceae), demonstrates significant anti-apoptotic effects in renal I/R injury (Liu et al., 2020). Their findings indicated that ellagic acid pretreatment reduced apoptosis both *in vivo* (rat I/R-AKI model) and *in vitro* (H/R-induced NRK-52E cells), as indicated by decreased TUNEL-positive cells, lower Bax and cleaved caspase-3 expression, and elevated Bcl-2. Mechanistically, ellagic acid inhibited the NOX4/JAK/STAT pathway, thereby suppressing oxidative stress and inflammation—key drivers of apoptosis. Notably, NOX4 overexpression reversed ellagic acid’s protective effects, underscoring its essential role in this mechanism.

#### 4.1.21 Sesamin

Sesamin, a bioactive lignan found in medicinal plants including *Sesamum indicum* L. (Pedaliaceae), protects against renal I/R injury by reducing apoptosis and inflammation (Li et al., 2016). Results from this research showed that, in a mouse model, sesamin pretreatment decreased tubular cell apoptosis, as shown by reduced TUNEL-positive cells and caspase-3 activity. It also suppressed pro-inflammatory cytokines like TNF-α and IL-1β and limited neutrophil infiltration. Mechanistically, sesamin enhanced the CD39-adenosine-A_2_AR pathway by upregulating CD39 and A_2_AR expression and increasing adenosine levels. These protective effects were partially reversed by CD39 inhibition or A_2_AR blockade, confirming the pathway’s role. This evidence indicates that sesamin alleviates renal I/R injury by modulating apoptosis and inflammation through adenosine signaling.

#### 4.1.22 Aloperine

Aloperine, a quinolizidine alkaloid found in medicinal plants including *Sophora alopecuroides* L. (Fabaceae), exerts notable anti-apoptotic effects in the context of renal I/R injury (Hu et al., 2016). The study demonstrated that pretreatment with aloperine significantly attenuated renal tubular apoptosis, supported by a reduction in TUNEL-positive cells and downregulation of caspase-3 expression in I/R-induced mice. Mechanistically, aloperine was found to enhance AP-1 transcriptional activity, which promoted the expression of the anti-apoptotic protein Bcl-2 and increased SOD levels, thereby alleviating oxidative stress and inhibiting apoptotic cell death. These results underscore the therapeutic potential of aloperine in modulating apoptosis during I/R-AKI.

#### 4.1.23 Other metabolites

In addition to the metabolites discussed above, many other bioactive TCM metabolites, including paeoniflorin, neferine, schisandrin B, resveratrol, costunolide, ganoderic acids, taraxasterol, chrysin, quercetin, diosmetin, oleanolic acid, and mangiferin, have demonstrated anti-apoptotic efficacy in I/R-AKI models. Their chemical structures, representative sources, experimental objects, specific molecular targets, and corresponding references are provided in Table 1.

### 4.2 Modulation of ferroptotic pathway

#### 4.2.1 Quercetin

Quercetin (QCT), a natural flavonoid found in various medicinal plants, notably *Styphnolobium japonicum* (L.) Schott (Fabaceae), has been shown to inhibit ferroptosis in renal TECs and alleviate I/R-AKI (Wang Y. et al., 2021). The study reported that *in vitro*, QCT counteracted erastin- or RSL3-induced ferroptosis in NRK-52E and HK-2 cells, improving cell survival while reducing lipid ROS and MDA and elevating GSH levels. Mechanistic studies indicated that QCT downregulated ATF3 expression, leading to subsequent upregulation of SLC7A11 and GPX4, which restored antioxidant capacity and suppressed ferroptosis. *In vivo*, QCT treatment attenuated renal damage and ferroptosis in I/R-AKI models, as demonstrated by reduced MDA, increased GSH, and restored GPX4 expression in kidney tissues. These results identify QCT as a promising therapeutic agent targeting ferroptosis in I/R-AKI.

#### 4.2.2 Paeoniflorin

Paeoniflorin (PF), a monoterpene glycoside isolated from medicinal plants such as *Paeonia lactiflora* Pall. (Paeoniaceae), exhibits renoprotective effects attributed to its anti-inflammatory, antioxidant, and immunomodulatory properties (Zhang and Wei, 2020). A recent study by Ma et al. specifically explored the potential of PF in attenuating ferroptosis in the context of I/R-AKI. Through both *in vivo* (murine bilateral RIR) and *in vitro* (HK2 cells subjected to H/R) models, the authors demonstrated that PF pretreatment dose-dependently improved renal function, reduced inflammatory cytokine levels, and suppressed key ferroptotic markers. Mechanistically, PF was shown to upregulate the expression of Slc7a11, the functional subunit of the System Xc^−^ cystine/glutamate antiporter. This upregulation enhanced GSH biosynthesis, which in turn restored the activity of GPX4 and diminished lethal lipid peroxidation. RNA sequencing analysis further confirmed that PF treatment reversed the H/R-induced suppression of Slc7a11 expression. Importantly, the protective effects of PF were abrogated in Slc7a11-knockdown cells, establishing that its anti-ferroptotic activity is strictly dependent on Slc7a11 (Ma et al., 2023). Collectively, these findings identify PF as a promising therapeutic agent against I/R-AKI by specifically targeting the Slc7a11/GPX4 axis to inhibit ferroptosis.

#### 4.2.3 Pachymic acid

Pachymic acid (PA), a lanostane-type triterpenoid which can be isolated from *Wolfiporia hoelen* (Fr.) Y.C.Dai and V. Papp (Fomitopsidaceae), exhibits renoprotective effects via anti-inflammatory and antioxidant properties. Jiang et al. demonstrated that PA pretreatment attenuates renal I/R injury in mice by inhibiting ferroptosis. PA dose-dependently improved renal function (reduced SCr and BUN) and ameliorated histological damage. It suppressed ferroptotic mitochondrial alterations, increased GSH, and decreased MDA and COX-2, indicating reduced lipid peroxidation. Moreover, PA upregulated GPX4, SLC7A11, and HO-1 expression, mediated through Nrf2 pathway activation. These findings highlight PA’s role in alleviating ferroptosis and its potential as a therapeutic agent for I/R-AKI (Jiang et al., 2021).

#### 4.2.4 Cyanidin-3-glucoside

Cyanidin-3-glucoside (C3G), a natural anthocyanin widely distributed in medicinal plants, notably *Morus alba* L. (Moraceae), with antioxidant and anti-inflammatory properties, effectively inhibits ferroptosis in renal TECs following I/R injury. Du et al. reported that C3G treatment significantly ameliorated both erastin-induced and H/R-induced ferroptosis in HK-2 cells and I/R-AKI mice, as evidenced by reduced lipid peroxidation (decreased MDA, 4-HNE, and lipid ROS), restored GSH levels, and regulation of key ferroptosis-related proteins (upregulation of GPX4 and downregulation of ACSL4). Mechanistically, C3G was shown to activate the AMPK signaling pathway. The use of the AMPK inhibitor Compound C (CC) or AMPK-specific siRNA abolished the protective effects of C3G, confirming that its anti-ferroptotic action is AMPK-dependent. Collectively, these results underscore the potential of C3G in mitigating renal I/R injury by targeting the AMPK-mediated ferroptosis pathway (Du et al., 2023).

#### 4.2.5 Salidroside

Salidroside (SA), a natural bioactive metabolite found in several medicinal plants such as *Rhodiola rosea* L. (Crassulaceae), alleviates renal I/R injury by targeting ferroptosis via PI3K/AKT pathway activation (Tang et al., 2023b). The study demonstrated that, in a rat model of I/R-AKI, pretreatment with SA significantly ameliorated renal tubular injury and improved renal function, as indicated by reduced levels of SCr and BUN. Mechanistically, SA inhibited oxidative stress, resulting in reduced accumulation of ROS and MDA, while enhancing the activity of antioxidant enzymes such as SOD and GSH. Furthermore, SA administration counteracted ferroptosis by upregulating GPX4 expression and downregulating ACSL4, thereby reducing lipid peroxidation. *In vitro* studies using H/R-treated NRK cells confirmed that SA exerted protective effects against ferroptosis, which were partially reversed by the PI3K/AKT pathway inhibitor PI3K/AKT-IN-1. These findings highlight the role of SA in mitigating renal I/R-induced ferroptosis via PI3K/AKT-mediated antioxidative mechanisms.

#### 4.2.6 Vaccarin

Obtained from the seeds of *Gypsophila vaccaria* (L.) Sm. (Caryophyllaceae), the flavonoid glycoside Vaccarin (VA) mitigates renal I/R injury through modulation of the ferroptotic pathway (Fan et al., 2025). The study found that this therapeutic effect was confirmed by its ability to markedly improve renal function (reduced SCr and BUN) and ameliorate histological damage in a mouse model of I/R-AKI. Mechanistically, VA exerted potent anti-ferroptotic effects by downregulating key markers of oxidative stress and lipid peroxidation, namely, ROS and MDA, while restoring GSH levels. Furthermore, VA administration reversed the I/R-induced suppression of central ferroptosis regulators, GPX4 and SLC7A11, thus restoring cellular antioxidant capacity and inhibiting iron-dependent lipid peroxidation. Although VA was shown to interact with NOX4 and reduce its expression, pharmacological inhibition or genetic modulation of NOX4 did not significantly alter the protective effects of VA, suggesting that its anti-ferroptotic action may involve multiple pathways beyond NOX4 inhibition.

#### 4.2.7 Echinocystic acid

The pentacyclic triterpene echinocystic acid (EA), a bioactive metabolite isolated from medicinal plants including *Gleditsia sinensis* Lam. (Fabaceae), has been shown to confer remarkable renoprotection in renal I/R injury through its anti-ferroptotic and anti-inflammatory properties (Dang et al., 2025). Their results showed that, using a neonatal rat model of I/R-AKI, it was observed that administration of EA markedly preserved renal function, as indicated by decreased serum concentrations of BUN and SCr, and attenuated histological kidney damage. Mechanistically, EA suppressed ferroptosis by decreasing levels of Fe^2+^, ROS, MDA, and myeloperoxidase (MPO), while enhancing antioxidant defenses through increased GSH, SOD, CAT, and GPx activities. Furthermore, EA downregulated pro-inflammatory cytokines (TNF-α, IL-1β, IL-6) and reduced macrophage infiltration (F4/80^+^cells), indicating anti-inflammatory effects. Importantly, EA activated the Nrf2/GPX4 pathway, and the protective effects were reversed by the Nrf2 inhibitor ML385, confirming that EA alleviates ferroptosis and renal injury primarily through Nrf2/GPX4 activation. Collectively, these results underscore the therapeutic potential of EA in I/R-AKI by modulating ferroptotic death and inflammatory responses.

#### 4.2.8 Loureirin C

Loureirin C (LC), a dihydrochalcone flavonoid isolated from several *Dracaena* species, notably *Dracaena angustifolia* (Medik.) Roxb. (Asparagaceae), exhibits considerable renoprotective properties in renal I/R injury through ameliorating mitochondrial impairment and ferroptotic cell death (Qi et al., 2024). Their research demonstrated that, in a mouse I/R-AKI model, administration of LC dose-dependently preserved renal function and reduced histological kidney damage. Mechanistically, LC suppressed ferroptosis by decreasing levels of Fe^2+^, MDA, and ROS, while enhancing the activity of antioxidant enzymes such as SOD and upregulating key anti-ferroptotic proteins GPX4 and Slc7a11. *In vitro*, using a H/R model in HK-2 cells, LC effectively scavenged mitochondrial ROS (mtROS), restored ATP production, improved mitochondrial respiration, and preserved mtDNA integrity. Furthermore, LC promoted the nuclear translocation of Nrf2 and activated the expression of downstream antioxidant genes HO-1 and NQO-1. The protective effects of LC were substantially reversed by either Nrf2 knockdown or pharmacological inhibition with ML385, confirming that LC alleviates oxidative stress and ferroptosis primarily through the Nrf2 pathway. Taken together, these results position LC as a promising TCM-associated agent capable of attenuating I/R-AKI via activation of the Nrf2 antioxidant axis and suppression of ferroptosis.

#### 4.2.9 Xanthohumol

Xanthohumol (XN), a prenylated flavonoid isolated from several medicinal plants, notably *Humulus lupulus* L. (Cannabaceae), demonstrates significant renoprotective effects against I/R-AKI by targeting ferroptosis and oxidative stress (Tang et al., 2023a). Investigators reported that in a rat model of renal I/R injury, XN administration ameliorated renal dysfunction (reduced BUN and SCr) and attenuated histopathological damage. Mechanistically, XN suppressed ferroptosis, as evidenced by reduced levels of MDA and ROS, while concurrently enhancing the antioxidant capacity through increased SOD activity and GSH levels. Furthermore, XN downregulated the pro-ferroptotic protein ACSL4 and upregulated the anti-ferroptotic protein GPX4. *In vitro*, in a H/R model of HK-2 cells, XN inhibited cell death and ferroptosis markers. The study identified that XN activates the Nrf2/HO-1 signaling pathway, and the protective effects of XN were significantly reversed by Nrf2 siRNA, confirming that XN exerts its anti-ferroptotic and antioxidant effects primarily through the Nrf2/HO-1 axis. These findings highlight XN as a potent natural metabolite capable of mitigating renal I/R injury by modulating ferroptosis and oxidative stress pathways.

#### 4.2.10 Silibinin

Silibinin, a bioactive flavonolignan isolated from *Silybum marianum* (L.) Gaertn (Asteraceae), has been widely recognized for its hepatoprotective effects, but emerging evidence suggests it also exerts renoprotective actions in I/R-AKI by targeting ferroptosis (Deng et al., 2024). In their study, Deng et al. showed that silibinin pretreatment significantly ameliorated renal dysfunction, pathological damage, and inflammation in a murine model of I/R-AKI. Mechanistically, silibinin inhibited ferroptosis both *in vivo* and *in vitro*, as evidenced by reduced lipid peroxidation (decreased MDA and increased GSH and SOD levels), diminished iron accumulation, and suppression of mitochondrial damage. Using HuProt human proteome microarray, the authors identified ferritin heavy chain 1 (FTH1) as a direct binding target of silibinin, which was further validated through molecular docking, surface plasmon resonance imaging (SPRI), cellular thermal shift assay (CETSA), and drug affinity responsive target stability (DARTS). Importantly, silibinin disrupted the NCOA4–FTH1 interaction, thereby inhibiting ferritinophagy and subsequent ferroptosis. Knockdown of FTH1 partially reversed the anti-ferroptotic effects of silibinin, confirming FTH1 as a critical mediator of its action. These findings highlight silibinin as a promising therapeutic agent for I/R-AKI through FTH1-targeted inhibition of ferroptosis.

### 4.3 Modulation of pyroptotic pathway

#### 4.3.1 Gypenoside XVII

Gypenoside XVII (GP-17), a tetracyclic triterpenoid saponin found in medicinal plants, notably *Gynostemma pentaphyllum* (Thunb.) Makino (Cucurbitaceae), has been demonstrated to attenuate renal I/R injury by targeting the pyroptotic pathway (Wang et al., 2024). Their results indicated that, in a mouse model of renal I/R injury, pretreatment with GP-17 significantly inhibited ERS, as evidenced by reduced expression of ERS-related proteins including BIP, p-PERK, and CHOP. This suppression of ERS further led to the inhibition of NLRP3 inflammasome activation, resulting in decreased cleavage of caspase-1 and GSDMD, key executors of pyroptosis. Consequently, GP-17 treatment reduced the release of pro-inflammatory cytokines such as IL-1β and IL-6, ameliorated renal tubular damage, and improved renal function markers (BUN and SCr). These findings suggest that GP-17 protects against I/R-AKI by mitigating ERS-mediated NLRP3 inflammasome activation and subsequent pyroptosis.

#### 4.3.2 Salvianolic acid B

Salvianolic acid B (SalB), a bioactive phenolic acid found in medicinal plants, notably *Salvia miltiorrhiza* Bunge (Lamiaceae), has been shown to exert protective effects against renal I/R injury by targeting pyroptosis (Pang et al., 2020). In that study, Pang et al. demonstrated that SalB pretreatment significantly ameliorated renal dysfunction and histopathological damage in mice, evidenced by reduced SCr and BUN levels. SalB effectively suppressed oxidative stress by enhancing the nuclear accumulation of Nrf2 and upregulating downstream antioxidant enzymes such as HO-1, thereby reducing ROS levels. Furthermore, SalB inhibited the activation of the NLRP3 inflammasome and subsequent caspase-1 cleavage, which led to decreased processing of GSDMD and IL-1β, key executors and mediators of pyroptosis. These effects were corroborated *in vitro* using H/R-treated HK-2 cells, where SalB reduced LDH release, pyroptotic cell death, and inflammasome assembly. The key role of Nrf2 was confirmed through siRNA knockdown experiments, which abolished SalB’s antioxidative and antipyroptotic effects. Thus, SalB attenuates pyroptosis in AKI primarily through the Nrf2-mediated suppression of oxidative stress and NLRP3 inflammasome activation.

#### 4.3.3 Berberine

Berberine (BBR), a natural isoquinoline alkaloid found in medicinal plants such as *C. chinensis* Franch. (Ranunculaceae), has demonstrated significant potential in attenuating pyroptosis in renal TECs under I/R conditions (Wang and Huang, 2025). Using an *in vitro* H/R model with HK-2 cells, Wang et al. showed in their research that BBR treatment significantly reduced the expression of key pyroptosis-related proteins, including NLRP3, caspase-1, IL-1β, and GSDMD-N. Mechanistically, BBR was found to activate the FOXO3a/ARC signaling pathway, as evidenced by increased phosphorylation of FOXO3a and upregulation of ARC expression. This activation correlated with reduced oxidative stress, decreased inflammatory cytokine release (TNF-α, IL-6, IL-1β), and improved cell viability. These findings suggest that BBR exerts its anti-pyroptotic effects in renal I/R injury at least partially through the FOXO3a/ARC axis, highlighting its potential as a therapeutic agent for mitigating pyroptosis in AKI.

#### 4.3.4 Naringenin

Naringenin (NRG), a natural flavonoid found in medicinal plants like *Citrus maxima* (Burm.) Merr. (Rutaceae), has shown significant protective effects against renal I/R injury by suppressing pyroptosis through modulation of ERS (Zhang et al., 2022). Their work revealed that, in both mouse I/R and HK-2 cell H/R models, NRG treatment markedly reduced the expression of key pyroptosis-related proteins, including NLRP3, ASC, caspase-1, GSDMD-N, caspase-11, and IL-1β. Mechanistically, NRG was found to inhibit ER stress by downregulating ERS markers such as GRP78, CHOP, and caspase-12. Furthermore, the activation of the Nrf2/HO-1 signaling pathway played a critical role in mediating these effects, as evidenced by the upregulated protein expression of both Nrf2 and its downstream target HO-1 following NRG administration. The use of brusatol, an Nrf2 inhibitor, reversed the protective effects of NRG, confirming the crucial involvement of the Nrf2/HO-1 axis. These results indicate that naringenin alleviates pyroptosis in renal I/R injury primarily by activating Nrf2/HO-1 signaling to suppress ER stress.

#### 4.3.5 Parthenolide

Parthenolide (PTL), a sesquiterpene lactone found in medicinal plants such as *Magnolia biondii* Pamp. (Magnoliaceae) (Schühly et al., 2009), has been identified as a specific NF-κB inhibitor and demonstrates protective effects against pyroptosis in renal I/R injury. In a study by Xiao et al., it was shown that Tisp40 overexpression in renal tubular epithelial cells (TCMK-1) exacerbated H/R-induced pyroptosis and promoted the phosphorylation of p65 (p-p65), a key subunit of NF-κB. Treatment with PTL effectively suppressed Tisp40-induced activation of the NF-κB pathway, as evidenced by reduced p-p65 levels. Consequently, PTL administration significantly attenuated NLRP3 inflammasome activation, caspase-1 cleavage, and the expression of GSDMD-N, ultimately reducing pyroptotic cell death. These findings indicate that parthenolide protects against renal I/R injury by inhibiting the NF-κB pathway, thereby suppressing GSDMD-mediated pyroptosis (Xiao et al., 2020).

### 4.4 Modulation of necroptotic pathway

#### 4.4.1 Aurantiamide

In the context of I/R-AKI, aurantiamide (AA), a bioactive metabolite found in medicinal plants including *Portulaca oleracea* L. (Portulacaceae) (Chen et al., 2016), has been shown to effectively attenuate renal injury by targeting the necroptotic pathway (He et al., 2024). The study by He et al. found that AA directly bound to the gastrin-releasing peptide receptor (GRPR), as was confirmed by molecular docking and cellular thermal shift assays (CETSA), and functioned as a novel GRPR antagonist. Through this mechanism, AA inhibited the phosphorylation of key necroptosis-related proteins, including RIPK1, RIPK3, and MLKL, thereby suppressing necroptosis in renal tubular epithelial cells under both *in vitro* H/R conditions and *in vivo* renal I/R injury models. Moreover, AA treatment significantly reduced expression of the renal injury marker KIM1 and downregulated inflammatory mediators such as MCP-1, IL-6, and TNF-α. Importantly, the renoprotective effects of AA were abolished in GRPR-knockout mice and GRPR-silenced cells, underscoring the essential role of GRPR in its mechanism of action. These findings highlighted AA as a promising TCM metabolite that modulates necroptosis via GRPR antagonism, offering a potential therapeutic strategy for I/R-AKI.

#### 4.4.2 Gypenoside XLIX

Gypenoside XLIX (Gyp XLIX), a dammarane-type saponin found in medicinal plants, notably *G. pentaphyllum* (Thunb.) Makino (Cucurbitaceae), has shown significant protective effects against renal necroptosis in I/R-AKI (Yang et al., 2021). Their research indicated that, in renal I/R injury models, Gyp XLIX treatment markedly reduced SCr and BUN levels, alleviated tubular damage, and suppressed the expression of KIM-1. Mechanistically, Gyp XLIX inhibited the phosphorylation of MLKL and suppressed the activation of the RIPK1/RIPK3 axis, thereby attenuating programmed necrotic cell death in renal tubular epithelial cells. Furthermore, RNA sequencing and molecular analyses revealed that Gyp XLIX downregulated insulin-like growth factor-binding protein 7 (IGFBP7) and reduced its binding to the IGF1 receptor (IGF1R), leading to enhanced IGF1R phosphorylation and activation of downstream survival signaling. The critical role of IGF1R was confirmed using the inhibitor picropodophyllin (PPP), which abolished the renoprotective effects of Gyp XLIX. Targeting the IGFBP7/IGF1R axis, Gyp XLIX emerges from this study as a promising TCM metabolite for suppressing necroptosis and inflammation in I/R-AKI.

## 5 RCD pathway crosstalk and evidence-based evaluation of TCM metabolites in I/R-AKI

The preceding sections have delineated the molecular mechanisms of four RCD pathways and catalogued TCM metabolites that modulate these RCD pathways. However, these pathways do not operate in isolation in I/R-AKI. A critical synthesis is therefore warranted to identify key molecular hubs of pathway crosstalk and to evaluate the strength of evidence supporting TCM metabolites as RCD modulators.

### 5.1 Key hubs of RCD pathway crosstalk

Several regulatory nodes mediate crosstalk among RCD pathways. Caspase-8, canonically associated with extrinsic apoptosis, also functions as a switch between apoptosis and necroptosis: when caspase-8 is inhibited, death receptor ligation drives necrosome assembly and necroptotic cell death instead (Hildebrand et al., 2015). The tumor suppressor p53 can promote apoptosis via PUMA/Bax (Roufayel et al., 2022) and also ferroptosis via SLC7A11 repression (Yuan et al., 2025). Notably, emodin and berberine inhibit p53 to attenuate apoptosis (Lin et al., 2018; Lu et al., 2023), yet whether they concurrently suppress ferroptosis in I/R-AKI through the same mechanism remains unexamined. Nrf2 serves as a master antioxidant hub whose activation can counteract ferroptosis, pyroptosis, and apoptosis (Zhang et al., 2019). Metabolites such as salvianolic acid B and naringenin are categorized as pyroptosis inhibitors through Nrf2 activation in Table 1 (Pang et al., 2020; Zhang et al., 2022); however, their Nrf2-dependent anti-ferroptotic potential in I/R-AKI has not been assessed. These examples suggest that metabolites categorized as single-pathway modulators may function as broad-spectrum RCD regulators—a hypothesis requiring systematic investigation.

### 5.2 Temporal hierarchy and dominant position of RCD pathways in I/R-AKI

The relative contributions and temporal hierarchy of distinct RCD pathways during I/R-AKI remain poorly defined and represent a critical knowledge gap. While ferroptosis has been shown to occur predominantly during the reperfusion phase (Tang et al., 2021), the precise sequence in which apoptosis, pyroptosis, and necroptosis are activated, and which pathway contributes most decisively to tubular injury and the subsequent transition to CKD, has not been systematically investigated. Direct, head-to-head comparisons employing selective inhibitors or genetic models within a single experimental system are urgently needed to determine the relative contributions of distinct RCD pathways. Meanwhile, time-course analyses are essential to resolve their temporal order at each stage of I/R-AKI.

### 5.3 Evidence-based evaluation of TCM metabolites

The Level A/B/C classification introduced in Section 4 enables a critical appraisal of the evidence base. Approximately half of the metabolites in Table 1 attain Level A, distinguished by rigorous orthogonal validation such as genetic knockout models, specific pathway inhibitor reversal, or target engagement assays; representative examples include polydatin (apoptosis), alisol B 23-acetate (apoptosis), aurantiamide (necroptosis), and silibinin (ferroptosis). Nonetheless, a comparable number of metabolites remain at Level B. Among these, some lack rigorous mechanistic validation beyond correlative protein expression changes, while a subset (including scutellarein, nicotiflorin, and eriocitrin) are supported by orthogonal mechanistic data but are retained at Level B owing to their recognized PAINS status. Few metabolites, including paeoniflorin (apoptosis), berberine (pyroptosis), and parthenolide (pyroptosis), are classified as Level C, with evidence derived exclusively from *in vitro* experiments. This evidence-tiered structure, therefore, not only marks high-risk metabolites but also highlights promising candidates for future clinical translation.

## 6 Conclusion, limitation and perspectives

### 6.1 Conclusion

This review consolidates compelling evidence that targeting RCD pathways—apoptosis, ferroptosis, pyroptosis, and necroptosis—represents a promising therapeutic strategy for I/R-AKI. We have detailed the distinct molecular mechanisms of these pathways in driving tubular injury and renal dysfunction and provided an integrative framework that aligns the four key RCD pathways with a spectrum of TCM metabolites that modulate them. While loganin and many other reviewed metabolites are not unique to TCM, they serve as important chemical constituents in numerous classic TCM, thus helping to clarify the pharmacological basis of TCM practice. Collectively, the preclinical evidence summarized in this review underscores the potential of TCM as a valuable repository of bioactive metabolites for renoprotection.

### 6.2 Limitation

Despite the promising findings synthesized herein, several limitations must be considered. First, the majority of the reviewed studies employed pretreatment protocols—administering metabolites before the onset of ischemia—whereas in clinical practice, I/R-AKI is largely unpredictable, limiting the translational relevance of these findings. Second, approximately half of the reviewed metabolites are supported by Level A evidence with rigorous orthogonal mechanistic validation, whereas others are supported primarily by correlative expression data or purely *in vitro* experiments. Additionally, findings concerning metabolites identified as PAINS in this review warrant cautious interpretation. Third, pharmacokinetic properties, including ADME (absorption, distribution, metabolism, and excretion) characteristics, bioavailability, and especially tissue-targeting profiles, are rarely examined in the reviewed studies. Fourth, the relative contributions and temporal hierarchy of distinct RCD pathways during I/R-AKI remain poorly defined. Finally, the lack of clinical validation of these preclinical findings underscores the translational gap that remains to be addressed.

### 6.3 Perspectives

To translate these preclinical findings into clinical benefit, future research should prioritize several key directions. First, the evaluation of TCM metabolites must shift from pretreatment protocols to clinically feasible post-ischemic or post-reperfusion administration, so as to better reflect the therapeutic window in real-world scenarios where the onset of I/R-AKI is largely unpredictable. Second, systematic pharmacokinetic characterization of promising metabolites—particularly those supported by Level A evidence—should be undertaken to address the current scarcity of bioavailability and tissue-targeting data. Formulation strategies such as nanoparticle encapsulation, prodrug design, and renal-targeted delivery systems may further help overcome the pharmacokinetic limitations of poorly bioavailable metabolites. Third, the relative contributions and temporal hierarchy of distinct RCD pathways during I/R-AKI need to be systematically elucidated using selective inhibitors, genetic models, and time-course analyses. In parallel, crosstalk among these pathways should be clarified through dedicated crosstalk analyses, thereby informing the rational design of multi-target metabolite combinations capable of modulating apoptosis, ferroptosis, pyroptosis, and necroptosis. Finally, bridging the translational gap will ultimately require clinical trials that evaluate the safety, pharmacokinetics, and efficacy of the promising TCM metabolites.

## Glossary

AA: aurantiamide
ABA: alisol B 23-acetate
ACSL4: acyl-CoA synthetase long-chain family member 4
AKI: acute kidney injury
ASC: apoptosis-associated speck-like protein containing a CARD
AS-IV: astragaloside IV
ATF3: activating transcription factor 3
BBR: berberine
BUN: blood urea nitrogen
C3G: cyanidin-3-glucoside
CHOP: C/EBP homologous protein
CKD: chronic kidney disease
CTS: cryptotanshinone
DAMPs: damage-associated molecular patterns
EA: echinocystic acid
ELE: β-elemene
ERS: endoplasmic reticulum stress
FTH1: ferritin heavy chain 1
FXR: Farnesoid X receptor
GFR: glomerular filtration rate
GP-17: gypenoside XVII
GPX4: glutathione peroxidase 4
GSDMD: gasdermin D
GSH: glutathione
Gyp XLIX: gypenoside XLIX
HMGB1: high-mobility group box 1
H/R: hypoxia/reoxygenation
HSYA: hydroxysafflor yellow A
I/R: ischemia/reperfusion
LC: loureirin C
LIP: labile iron pool
MA: madecassoside
MDA: malondialdehyde
MLKL: mixed lineage kinase domain-like pseudokinase
NR1: notoginsenoside R1
NRG: naringenin
PA: pachymic acid
PAINS: pan-assay interference compounds
PF: paeoniflorin
PTL: parthenolide
PUFA: polyunsaturated fatty acid
QCT: quercetin
RCD: regulated cell death
RIPK1: receptor-interacting serine/threonine-protein kinase 1
RIPK3: receptor-interacting serine/threonine-protein kinase 3
SA: salidroside
SalB: salvianolic acid B
SCr: serum creatinine
Shh: Sonic hedgehog
SOD: superoxide dismutase
TCM: traditional Chinese medicine
TECs: tubular epithelial cells
TMP: tetramethylpyrazine
VA: vaccarin
XN: xanthohumol.
