# Supplementary material for: Modulating regulated cell death: mechanistic insights into traditional Chinese medicine metabolites for ischemia/reperfusion-induced acute kidney injury
Source: Front Pharmacol. 2026 Jul 2;17:1779205. doi: 10.3389/fphar.2026.1779205 (PMC13373590; doi:10.3389/fphar.2026.1779205)
Supplement: Supplementary file 1 [file Table1.docx]

**Supplemental Table S1.** Detailed experimental parameters of TCM metabolites in preclinical studies of I/R-AKI.

| **Metabolite** | **In vivo study** | | | **In vitro study** | | | **Type of material** | **Reference** |
| --- | --- | --- | --- | --- | --- | --- | --- | --- |
|  | **Model** | **Dose & regimen** | **Controls** | **Model** | **Dose & duration** | **Controls** |  |  |
| Loganin | Mice; I/R  30 min/24 h | 5, 10, 20 mg/kg, i.p., once daily for 7 days before ischemia;  5 mg/kg (lowest protective dose tested) | Sham; I/R; Positive control: None | NRK-52E cells; H/R  12 h/12 h | 10, 20, 40 μM; 4 h prior to H/R  Treatment;  10 μM (lowest protective dose tested) | Normoxia; H/R; Positive control: None | Single metabolite | (Huang et al., 2022) |
| Astragaloside IV (AS-IV) | Rats; I/R  45 min/24 h | 2, 5, 10 mg/kg, i.g., single dose 1 h before ischemia;  5 mg/kg (lowest protective dose tested) | Sham;  I/R+vehicle; Positive control: None | **—** | **—** | **—** | Single metabolite | (Su et al., 2022) |
| Astragaloside IV (AS-IV) | Rats; I/R  45 min/24 h | 20 mg/kg, i.g., once daily for 7 days before ischemia | Sham+saline; I/R+saline; I/R+vehicle; Positive control: None | **—** | **—** | **—** | Single metabolite | (Gui et al., 2013) |
| Emodin | Mice; I/R  35 min/24 h | 1, 3, 10 mg/kg, i.p., once daily for 7 days before ischemia;  1 mg/kg (lowest protective dose tested) | Sham+vehicle; I/R+vehicle; Positive control: None | HK‑2 cells; H/R  12 h/8 h | 0.3, 1, 3 μM; 24 h co‑incubation prior to H/R;  1 μM (lowest protective dose tested) | Normoxia; H/R; Positive control: None | Single metabolite | (Wang et al., 2022b) |
| Emodin | Rats; I/R  45min/24h | 30 mg/kg, i.g., once daily for 7 days before ischemia | Sham+vehicle; I/R+vehicle; Positive control: None | HK‑2 cells; H/R (sealed 5h) | 10, 30, 50, 80 μM; treatment for 4–48 h after H/R;  10 μM (lowest protective dose tested) | Normoxia; H/R; Positive control: None | Single metabolite | (Lu et al., 2023) |
| Cordycepin | Rats; I/R  45 min/24 h | 2, 4, 8 mg/kg, i.g., once daily for 7 days before ischemia;  2 mg/kg (lowest protective dose tested) | Sham+vehicle; I/R+vehicle; Positive control: None | **—** | **—** | **—** | Single metabolite | (Han et al., 2020) |
| Ligustrazine | Rats; I/R  50 min/24 h | 40 mg/kg, i.p., every 6 h during reperfusion | Sham; I/R; Positive control: None | NRK-52E cells; CoCl₂ (500 μM, 12 h) or H/R  2 h/24 h | 30, 50 μM; 24 h treatment after insult;  30 μM (lowest protective dose tested) | Normoxia; CoCl₂ or H/R model; Positive control: None | Single metabolite | (Jiang et al., 2020) |
| Ligustrazine | Mice; I/R  50 min/24 h | 80 mg/kg, i.p., 30 min before ischemia | Sham+vehicle; I/R+vehicle; Positive control: None | **—** | **—** | **—** | Single metabolite | (Feng et al., 2011) |

**Supplemental Table S1.** (Continued) Detailed experimental parameters of TCM metabolites in preclinical studies of I/R-AKI.

| **Metabolite** | **In vivo study** | | | **In vitro study** | | | **Type of material** | **Reference** |
| --- | --- | --- | --- | --- | --- | --- | --- | --- |
|  | **Model** | **Dose & regimen** | **Controls** | **Model** | **Dose & duration** | **Controls** |  |  |
| Ligustrazine | Mice; I/R  50 min/24 h | 80 mg/kg, i.p.,  30 min before ischemia | Sham;  I/R+vehicle; Positive control: None | **—** | **—** | **—** | Single metabolite | **(**Feng et al., 2004) |
| Berberine (BBR) | **—** | **—** | **—** | HK‑2 cells; H/R  24 h/12 h | 10, 25, 50, 75, 100 μM;  2 h pretreatment before H/R; 10 μM (lowest protective dose tested) | Normoxia; Normoxia+BBR; H/R; Positive control: None | Single metabolite | (Yu et al., 2013) |
| Berberine (BBR) | Rats; I/R  45 min/6 h or  45 min/24 h | BBR: 2, 4 mg/kg; BBR-NP: 2, 4 mg/kg; tail vein injection, after ischemia;  2 mg/kg (lowest protective dose tested) | I/R+vehicle; I/R+blank NP; Positive control: None | **—** | **—** | **—** | Nanoparticle formulation vs. free single metabolite | (Xie et al., 2017) |
| Berberine (BBR) | **—** | **—** | **—** | NRK-52E cells; H/R  6 h/2 h | 10, 30, 60, 90 μM; 2 h pretreatment before H/R;  10 μM (lowest protective dose tested) | Normoxia; H/R; Positive control: None | Single metabolite | (Lin et al., 2018) |
| Cryptotanshinone (CTS) | Mice; I/R  30 min/24 h | 10 mg/kg/d, i.p., for 7 days prior to I/R | Sham+vehicle; Sham+CTS;  I/R+vehicle; Positive control: None | **—** | **—** | **—** | Single metabolite | (Bai et al., 2019) |
| Cryptotanshinone (CTS) | **—** | **—** | **—** | HK‑2 cells; H/R  12 h/2 h | 10, 20, 40 μM; 12 h pretreatment before H/R; 10 μM (lowest protective dose tested) | Normoxia; H/R; Positive control: None | Single metabolite | (Zhu et al., 2019) |
| Hyperoside | Mice; I/R  30 min/24 h | 20 mg/kg, i.p., 2 h prior to I/R | Sham; I/R; Positive control: None | HK‑2 cells; CoCl₂ (300 μM, 12 h) | 50, 100, 150, 200 μM; 1 h pretreatment before CoCl₂; 50 μM (lowest protective dose tested) | Normoxia; CoCl₂; Positive control: None | Single metabolite | (Wu et al., 2019) |

**Supplemental Table S1.** (Continued) Detailed experimental parameters of TCM metabolites in preclinical studies of I/R-AKI.

| **Metabolite** | **In vivo study** | | | **In vitro study** | | | **Type of material** | **Reference** |
| --- | --- | --- | --- | --- | --- | --- | --- | --- |
|  | **Model** | **Dose & regimen** | **Controls** | **Model** | **Dose & duration** | **Controls** |  |  |
| Hydroxysafflor yellow A (HSYA) | Mice; I/R  30 min/24 h | 100 mg/kg/d, i.p., for 7 consecutive days before I/R | Sham;  I/R+vehicle; Positive control: None | HK‑2 cells; H/R  12 h/2 h | 5, 10, 20 μg/ml; 12 h pretreatment before H/R;  5 μg/ml (lowest protective dose tested) | Normoxia; H/R; Positive control: None | Single metabolite | (Wang et al., 2022a) |
| Notoginsenoside R1  (NR1) | Rats; I/R  45 min/72 h | 20, 40 mg/kg/d, i.p., 1 h before ischemia, then once daily until sacrifice;  20 mg/kg/d (lowest protective dose tested) | Sham;  I/R+vehicle; Positive control: None | **—** | **—** | **—** | Single metabolite | (Liu et al., 2010) |
| Polydatin | Mice; I/R  30 min/72 h | 40 mg/kg, i.p.,  1 h before ischemia, then daily for 2 days | Sham; I/R+vehicle;  Positive control: None | Primary TECs; H/R (2, 4, or 6 h) / 24 h | 10, 20, 40 μM;  30 min before H/R  to end of reoxygenation; 20 μM (lowest protective dose tested) | Normoxia; H/R+vehicle;  Positive control: None | Single metabolite | (Meng et al., 2016) |
| Alisol B 23-acetate  (ABA) | Mice; I/R  35 min/24 h | 60 mg/kg/d, i.p., for 4 days before ischemia | Sham+vehicle; I/R+vehicle; Sham+ABA; Positive control: None | mTECs; mIMCD3; HEK293T (FXR reporter assay) | 5 μM, 24 h; Various concentrations for FXR activation assay | Vehicle; Positive control: CDCA | Single metabolite | (Luan et al., 2021) |
| Tetramethylpyrazine  (TMP) | Rats; I/R  45 min/24 h | 40 mg/kg, i.p., at 6 h intervals immediately after reperfusion | Sham; I/R; Positive control: None | NRK cells; H/R  6 h/18 h | 50 μM, during reoxygenation | Normoxia; H/R; Positive control: None | Single metabolite | (Sun et al., 2020) |
| β-elemene (ELE) | Mice; I/R  45 min/24 h | 40 mg/kg/d, i.p., for 7 days before ischemia | Sham;  Sham+ELE; I/R; Positive control: None | NRK52E cells; H₂O₂ (600 μM,  6 h) | 5, 10, 20 μM; pretreatment before H₂O₂; 5 μM (lowest protective dose tested) | Control; H₂O₂; Positive control: None | Single metabolite | (Gong et al., 2025) |
| Madecassoside (MA) | Mice; I/R  40 min/24 h | 40 mg/kg, i.p., 12 h before ischemia | Sham+vehicle; I/R+vehicle; Positive control: None | mTECs; H/R  12 h/6 h | 4 μM; 12 h pretreatment before hypoxia | Normoxia; H/R; Positive control: None | Single metabolite | (Shan et al., 2024) |

**Supplemental Table S1.** (Continued) Detailed experimental parameters of TCM metabolites in preclinical studies of I/R-AKI.

| **Metabolite** | **In vivo study** | | | **In vitro study** | | | **Type of material** | **Reference** |
| --- | --- | --- | --- | --- | --- | --- | --- | --- |
|  | **Model** | **Dose & regimen** | **Controls** | **Model** | **Dose & duration** | **Controls** |  |  |
| Tilianin | Mice; I/R  35 min/24 h | 5, 10, 15 mg/kg, i.g., once daily for 7 days before ischemia; 5 mg/kg (lowest protective dose tested) | Control; Sham; I/R;  I/R+vehicle; Positive control: None | **—** | **—** | **—** | Single metabolite | (Liu et al., 2022b) |
| Scutellarein | Rats; I/R  30 min/48 h | 5, 10, 20 mg/kg, i.g., every other day for 20 consecutive days before I/R;  5 mg/kg (lowest protective dose tested) | Sham; I/R; Positive control: None | HK‑2 cells; H/R  24 h/6 h | 2.5, 5, 10 μM; 24 h pretreatment before H/R; 2.5 μM (lowest protective dose tested) | Normoxia; H/R+vehicle;  Positive control: None | Single metabolite | (Liu et al., 2021) |
| Nicotiflorin | Mice; I/R  45 min/24 h | 50 mg/kg, i.g., once daily for 6 days before I/R | Control;  Sham; I/R; I/R+vehicle; Positive control: None | HK‑2 cells; H/R  24 h/6 h | 75 μg/ml; pretreatment before H/R | Normoxia; H/R+vehicle;  Positive control: None | Single metabolite | (Wang et al., 2021a) |
| Eriocitrin | Rats; I/R  1 h/24 h | 15, 30, 60 mg/kg, i.p., once daily from the day of modeling; 15 mg/kg (lowest protective dose tested) | Sham; I/R; Positive control: None | HK‑2 cells; H/R  1.5 h/6 h | 1, 2, 4 μM; pre- and  co-treatment with OGD/R; 1 μM (lowest protective dose tested) | Normoxia; H/R; Positive control: None | Single metabolite | (Xu et al., 2021) |
| Ellagic acid | Rats;  I/R 45 min/  60 min | 50, 100, 150 mg/kg, i.g., 30 min before ischemia; 50 mg/kg (lowest protective dose tested) | Sham; I/R; Positive control: None | NRK-52E cells; H/R  24 h/12 h | 5, 10, 20 μM; 24 h pretreatment before H/R; 5 μM (lowest protective dose tested) | Normoxia; H/R; Positive control: None | Single metabolite | (Liu et al., 2020) |
| Sesamin | Mice; I/R  30 min/24 h | 100 mg/kg, i.g., every 8 h for 3 times within 24 h before I/R | Sham; I/R+vehicle; Positive control: None | **—** | **—** | **—** | Single metabolite | (Li et al., 2016) |
| Aloperine | Mice; I/R  45 min/24 h | 50 mg/kg, i.g., once daily for 8 consecutive days before I/R | Sham; Sham+Alo;  I/R+vehicle; Positive control: None | HK‑2 cells; H/R  3 h/24 h | 0.5 mmol/L; present during hypoxia | Normoxia; H/R; Positive control: None | Single metabolite | (Hu et al., 2016) |

**Supplemental Table S1.** (Continued) Detailed experimental parameters of TCM metabolites in preclinical studies of I/R-AKI.

| **Metabolite** | **In vivo study** | | | **In vitro study** | | | **Type of material** | **Reference** |
| --- | --- | --- | --- | --- | --- | --- | --- | --- |
|  | **Model** | **Dose & regimen** | **Controls** | **Model** | **Dose & duration** | **Controls** |  |  |
| Paeoniflorin | **—** | **—** | **—** | HK-2 cells; H/R induced by CoCl₂,  24 h/4 h | 50, 100, 200 μM; 4 h pretreatment before H/R;  50 μM (lowest protective dose tested) | Normoxia; H/R; Positive control: None | Single metabolite | (Xing et al., 2023) |
| Neferine | Mice; I/R  30 min/24 h | 20 mg/kg/d, i.p., for 3 days before I/R | Control+vehicle; Control+Neferine; I/R; I/R+vehicle; Positive control: None | NRK-52E cells; hypoxia (antimycin A, 2 h) | 2, 4, 8 μM; 2 h pretreatment before H/R; 2 μM (lowest protective dose tested) | Normoxia; H/R; Positive control: None | Single metabolite | (Li et al., 2019) |
| Schisandrin B | Mice; I/R  45 min/24 h | 20, 40 mg/kg/d, i.g., for 7 consecutive days before ischemia;  20 mg/kg/d (lowest protective dose tested) | Sham+vehicle; I/R+vehicle; Positive control: None | HK‑2 cells; H/R  12 h/2 h | 5, 10, 15, 20 μM; 12 h pretreatment before hypoxia;  10 μM (lowest protective dose tested) | Normoxia; H/R; Positive control: None | Single metabolite | (Xu et al., 2025) |
| Resveratrol | Rats; I/R  30 min/2 h | 30 mg/kg, i.p.,  30 min before ischemia | Sham; I/R;  I/R+vehicle; Positive control: None | **—** | **—** | **—** | Single metabolite | (Alaasam et al., 2024) |
| Costunolide | Rats; I/R  45 min/24 h | 5, 10 mg/kg, i.p., 30 min before reperfusion;  5 mg/kg (lowest protective dose tested) | Sham; I/R; Positive control: None | **—** | **—** | **—** | Single metabolite | (Güler et al., 2023) |
| Ganoderic Acids (GAs) | Mice; I/R  35 min/24 h  or 48 h | 1.7, 5, 15 mg/kg/d, i.p., for 3 days before I/R until sacrifice;  1.7 mg/kg/d (lowest protective dose tested) | Sham+vehicle;  Sham+GAs; I/R+vehicle; Positive control: None | NRK-52E cells; H/R  12 h/12 h | 3.125, 12.5, 50 μg/ml; 12 h co-incubation before H/R; 3.125 μg/ml (lowest protective dose tested) | Normoxia; H/R; Positive control: None | Single metabolite | (Shao et al., 2021) |

**Supplemental Table S1.** (Continued) Detailed experimental parameters of TCM metabolites in preclinical studies of I/R-AKI.

| **Metabolite** | **In vivo study** | | | **In vitro study** | | | **Type of material** | **Reference** |
| --- | --- | --- | --- | --- | --- | --- | --- | --- |
|  | **Model** | **Dose & regimen** | **Controls** | **Model** | **Dose & duration** | **Controls** |  |  |
| Taraxasterol | Mice; I/R  30 min/3 d  or 14 d | 5, 10 mg/kg, i.p., starting 2 h before ischemia, then every 24 h;  5 mg/kg (lowest protective dose tested) | Sham+vehicle; I/R+vehicle; Positive control: Astragaloside IV | HK‑2 cells; H/R  12 h/2 h | 5, 10 μM; pretreatment before hypoxia; 5 μM (lowest protective dose tested) | Normoxia+  vehicle; H/R+vehicle; Positive control: Astragaloside IV | Single metabolite | (Li et al., 2020) |
| Chrysin | Mice; I/R  30 min/48 h | 100 mg/kg, i.p., once daily for 3 days before I/R | Sham+vehicle; I/R+vehicle; Positive control: None | **—** | **—** | **—** | Single metabolite | (Xu et al., 2019 |
| Quercetin (QCT) | Rats; I/R  30 min/5 h | 100 mg/kg, single dose, i.g. or i.p., 1 h before ischemia | Sham; I/R; Positive control: None | **—** | **—** | **—** | Single metabolite | (Bagheri et al., 2023) |
| Diosmetin | Mice; I/R  45 min/24 h | 0.25, 0.5, 1 mg/kg, i.p., 45 min before ischemia;  0.25 mg/kg (lowest protective dose tested) | Sham+vehicle; I/R+vehicle; Positive control: None | **—** | **—** | **—** | Single metabolite | (Yang et al., 2017) |
| Oleanolic acid | Rats; I/R  45 min/6 h | 12.5, 25, 50 mg/kg, i.p., once daily for 15 consecutive days before ischemia;  12.5 mg/kg (lowest protective dose tested) | Sham; I/R; Positive control: None | **—** | **—** | **—** | Single metabolite | (Long et al., 2016) |
| Mangiferin | Mice; I/R  30 min/24 h | 10, 30, 100 mg/kg/d, i.g., for 7 days before I/R until sacrifice;  100 mg/kg/d (lowest protective dose tested) | Sham+vehicle; Sham+Mangiferin; I/R+vehicle; Positive control: None | **—** | **—** | **—** | Single metabolite | (Wang et al., 2015) |

**Supplemental Table S1.** (Continued) Detailed experimental parameters of TCM metabolites in preclinical studies of I/R-AKI.

| **Metabolite** | **In vivo study** | | | **In vitro study** | | | **Type of material** | **Reference** |
| --- | --- | --- | --- | --- | --- | --- | --- | --- |
|  | **Model** | **Dose & regimen** | **Controls** | **Model** | **Dose & duration** | **Controls** |  |  |
| Quercetin (QCT) | Mice; I/R  30 min/24 h | 25 mg/kg, i.g., 3 times/day starting at model induction (8 h intervals) | Sham; I/R; Positive control: Fer-1 | NRK-52E cells; Erastin (1 μM, 24 h) or RSL3 (0.5 μM, 24 h) | 10 μM;  co-treatment with ferroptosis inducer for 24 h | Normal control; Erastin or RSL3; Positive control: Fer-1 | Single metabolite | (Wang et al., 2021b) |
| Paeoniflorin (PF) | Mice; I/R  40 min/24 h | 25, 50, 100 mg/kg, i.p., once daily for 3 days before I/R;  25 mg/kg (lowest protective dose tested) | Sham; Sham+PF; I/R+vehicle; Positive control: Fer-1 | HK‑2 cells; H/R  24 h/3 h | 25, 50, 100μM; pretreatment before H/R; 25 μM (lowest protective dose tested) | Normoxia;  Normoxia+PF; H/R; Positive control: Fer-1 | Single metabolite | (Ma et al., 2023) |
| Pachymic acid (PA) | Mice; I/R  40 min/24 h | 5, 10, 20 mg/kg, i.p., once daily for 3 days before I/R;  10 mg/kg (lowest protective dose tested) | Sham+vehicle; I/R+vehicle; Positive control: None | **—** | **—** | **—** | Single metabolite | (Jiang et al., 2021) |
| Cyanidin-3-glucoside (C3G) | Mice; I/R  33 min/24 h | 10 mg/kg, i.p., once daily for 1 week before I/R | Sham+vehicle; Sham+C3G; I/R+vehicle; Positive control: None | HK‑2 cells; H/R  6 h/24 h; Erastin (1 μM, 24 h) | 50 μM; 12 h pretreatment before hypoxia; co-treatment with Erastin for 24 h | Normoxia;  Normoxia+C3G; H/R; Positive control: None.  Normal control; Erastin; Positive control: Lip-1 | Single metabolite | (Du et al., 2023) |
| Salidroside (SA) | Rats; I/R  45 min/24 h | 1, 10, 100 mg/kg, i.g., once daily for 7 days before I/R;  1 mg/kg (lowest protective dose tested) | Sham; I/R; Positive control: None | NRK cells; H/R  8 h/12 h | 1, 10, 100, 1000 μM; added at reoxygenation; 1 μM (lowest protective dose tested) | Normoxia; H/R; Positive control: None | Single metabolite | (Tang et al., 2023b) |
| Vaccarin (VA) | Mice; I/R  40 min/24 h | 25 mg/kg, i.p., at 2 h and 12 h after reperfusion | Sham+vehicle; Sham+VA; I/R; Positive control: None | mTECs; H/R (12 h hypoxia) | 10, 20, 40, 80, 160 μM; optimal 40 μM; timing unspecified | Normoxia;  Normoxia+VA; H/R; Positive control: None | Single metabolite | (Fan et al., 2025) |

**Supplemental Table S1.** (Continued) Detailed experimental parameters of TCM metabolites in preclinical studies of I/R-AKI.

| **Metabolite** | **In vivo study** | | | **In vitro study** | | | **Type of material** | **Reference** |
| --- | --- | --- | --- | --- | --- | --- | --- | --- |
|  | **Model** | **Dose & regimen** | **Controls** | **Model** | **Dose & duration** | **Controls** |  |  |
| Echinocystic acid (EA) | Neonatal rats; I/R  45 min/48 h | 20, 40 mg/kg, i.p., at 24 h after I/R; 20 mg/kg (lowest protective dose tested) | Sham+vehicle; I/R; Positive control: None | **—** | **—** | **—** | Single metabolite | (Dang et al., 2025) |
| Loureirin C (LC) | Mice; I/R  45 min/24 h | 10, 25, 50 mg/kg, i.g., once daily for 3 days before I/R;  10 mg/kg (lowest protective dose tested) | Sham; I/R;  I/R+vehicle; Positive control: None | HK‑2 cells; H/R  24 h/12 h | 0.1, 0.5, 5, 10, 20μM; pretreatment for 24h before H/R;  0.5 μM (lowest protective dose tested) | Normoxia; H/R; HR+vehicle; Positive control: None | Single metabolite | (Qi et al., 2024) |
| Xanthohumol (XN) | Rats; I/R  45 min/24 h | 0.4 mg/kg, i.p., 10 min before I/R | Sham; I/R; Positive control: None | HK‑2 cells; H/R (8 h hypoxia) | 10 μM;  added at reoxygenation | Normoxia; H/R; Positive control: None | Single metabolite | (Tang et al., 2023a) |
| Silibinin | Mice; I/R  30 min/24 h | 25, 50, 100 mg/kg, i.p., once daily for 3 days before I/R;  25 mg/kg (lowest protective dose tested) | Sham+vehicle; I/R+vehicle; Positive control: Fer-1 | HK-2 / NRK-52E cells; Erastin (20 μM, 24 h) | 50 μM; co-treatment with ferroptosis inducer | Normal control; Erastin; Positive control: Fer-1 | Single metabolite | (Deng et al., 2024) |
| Gypenoside XVII (GP-17) | Mice; I/R  40 min/24 h | 10, 20, 40 mg/kg, i.g., once daily for 7 days before I/R;  10 mg/kg (lowest protective dose tested) | Sham+vehicle; I/R+vehicle; Positive control: 4-PBA | **—** | **—** | **—** | Single metabolite | (Wang et al., 2024) |
| Salvianolic acid B (SalB) | Mice; I/R  40 min/24 h | 50, 100, 200 mg/kg, i.g., once daily for 7 days before I/R;  50 mg/kg (lowest protective dose tested) | Sham+vehicle; I/R; Positive control: None | HK‑2 cells; H/R  6 h/1 h | 1, 5, 10, 20, 40, 80 μM; 24 h pretreatment before HR; 20 μM (lowest protective dose tested) | Normoxia; H/R; Positive control: MCC950,  VX-765 | Single metabolite | (Pang et al., 2020) |

**Supplemental Table S1.** (Continued) Detailed experimental parameters of TCM metabolites in preclinical studies of I/R-AKI.

| **Metabolite** | **In vivo study** | | | **In vitro study** | | | **Type of material** | **Reference** |
| --- | --- | --- | --- | --- | --- | --- | --- | --- |
|  | **Model** | **Dose & regimen** | **Controls** | **Model** | **Dose & duration** | **Controls** |  |  |
| Berberine (BBR) | **—** | **—** | **—** | HK‑2 cells; H/R  24 h/12 h | 1, 5, 10, 20, 40 μM; pretreatment before H/R,  1 μM (lowest protective dose tested) | Normoxia;  Normoxia+BBR; H/R; Positive control: None | Single metabolite | (Wang and Huang, 2025) |
| Naringenin (NRG) | Mice; I/R  30 min/24 h | 50 mg/kg/d, i.g., for 3 days before I/R | Sham; I/R;  I/R+vehicle; Positive control: 4-PBA | HK‑2 cells; H/R  12 h/4 h | 200 μM; added 24 h before H/R | Normoxia; H/R; H/R+vehicle; Positive control: 4-PBA | Single metabolite | (Zhang et al., 2022) |
| Parthenolide (PTL) | **—** | **—** | **—** | TCMK-1 cells; H/R  24 h/6 h | Concentration not specified; co-treatment with H/R | TCMK-1/vector; TCMK-1/Tisp40; Positive control: None | Single metabolite | (Xiao et al., 2020) |
| Aurantiamide (AA) | Mice; I/R  40 min/24 h | 2.5, 5, 10 mg/kg/d, i.g., for 3 days before I/R;  2.5 mg/kg (lowest protective dose tested) | Sham; Sham+AA; I/R; Positive control: RH-1402 | HK‑2 cells; H/R  12 h/6 h | 25, 50, 100μM; pretreatment before H/R (timing not specified);  25 μM (lowest protective dose tested) | Normoxia;  Normoxia+AA; H/R;  Positive control: RH-1402 | Single metabolite | (He et al., 2024) |
| Gypenoside XLIX (Gyp XLIX) | Mice; I/R  40 min/24 h | 25, 50, 100 mg/kg, i.p., 6 h before I/R, then once daily;  25 mg/kg (lowest protective dose tested) | Sham;  Sham+Gyp; I/R; Positive control: Curcumin | HK‑2 cells; H/R  12 h/6 h | 64 μM; pretreatment before H/R (timing not specified) | Normoxia;  Normoxia+Gyp; H/R; Positive control: None | Single metabolite | (Yang et al., 2021) |
